# The draft genome sequence of the spider *Dysdera silvatica* (Araneae, Dysderidae): A valuable resource for functional and evolutionary genomic studies in chelicerates

**DOI:** 10.1093/gigascience/giz099

**Published:** 2019-08-20

**Authors:** Jose Francisco Sánchez-Herrero, Cristina Frías-López, Paula Escuer, Silvia Hinojosa-Alvarez, Miquel A Arnedo, Alejandro Sánchez-Gracia, Julio Rozas

**Affiliations:** 1 Departament de Genètica, Microbiologia i Estadística, Universitat de Barcelona (UB) and Institut de Recerca de la Biodiversitat (IRBio), Diagonal 643, 08028 Barcelona, Spain; 2 Jardín Botánico, Instituto de Biología, Universidad Nacional Autónoma de México, Tercer Circuito Exterior S/N, Ciudad Universitaria Coyoacán, 04510 México DF, México; 3 Departament de Biologia Evolutiva, Ecologia i Ciències Ambientals, Universitat de Barcelona (UB) and Institut de Recerca de la Biodiversitat (IRBio), Diagonal 643, 08028 Barcelona, Spain

**Keywords:** Araneomorphae, hybrid genome assembly, genome annotation, Canary Islands

## Abstract

**Background:**

We present the draft genome sequence of *Dysdera silvatica*, a nocturnal ground-dwelling spider from a genus that has undergone a remarkable adaptive radiation in the Canary Islands.

**Results:**

The draft assembly was obtained using short (Illumina) and long (PaciBio and Nanopore) sequencing reads. Our *de novo* assembly (1.36 Gb), which represents 80% of the genome size estimated by flow cytometry (1.7 Gb), is constituted by a high fraction of interspersed repetitive elements (53.8%). The assembly completeness, using BUSCO and core eukaryotic genes, ranges from 90% to 96%. Functional annotations based on both *ab initio* and evidence-based information (including *D. silvatica* RNA sequencing) yielded a total of 48,619 protein-coding sequences, of which 36,398 (74.9%) have the molecular hallmark of known protein domains, or sequence similarity with Swiss-Prot sequences. The *D. silvatica* assembly is the first representative of the superfamily Dysderoidea, and just the second available genome of Synspermiata, one of the major evolutionary lineages of the “true spiders” (Araneomorphae).

**Conclusions:**

Dysderoids, which are known for their numerous instances of adaptation to underground environments, include some of the few examples of trophic specialization within spiders and are excellent models for the study of cryptic female choice. This resource will be therefore useful as a starting point to study fundamental evolutionary and functional questions, including the molecular bases of the adaptation to extreme environments and ecological shifts, as well of the origin and evolution of relevant spider traits, such as the venom and silk.

## Data Description

Spiders are a highly diverse and abundant group of predatory arthropods, found in virtually all terrestrial ecosystems. Approximately 45,000 spider species have been recorded to date [1]. The nocturnal ground family Dysderidae ranks 17th out of 118 currently accepted spider families in number of species. The type genus of the family, *Dysdera* Latreille, 1804, includes half of the family diversity (282 species). This genus is remarkable in several aspects. First, it represents one of the few cases of stenophagy, i.e., prey specialization, across spiders [2]. Many species in the genus have evolved special morphological, behavioral, and physiological adaptations to feed on woodlice, including modifications of mouthparts, unique hunting strategies, and effective restriction to assimilation of metals into its tissues [3–7]. Because of their chemical defenses and ability to accumulate heavy metals from the soil, woodlice are usually avoided as prey by most spiders, including generalist *Dysdera* [2,4, 5,7]. Although mostly circumscribed to the Mediterranean region, *Dysdera* has colonized all the Macaronesian archipelagoes and has undergone a remarkable species diversification in the Canary Islands [8]. As many as 55 species have been recorded across the 7 main islands and islets of this archipelago, being most of them single-island endemics [9]. Although multiple colonization events may account for the initial origin of species diversity the bulk of this diversity is the result of *in situ* diversification [8]. *Dysdera* spiders have adapted to a broad range of terrestrial habitats within the Canary Islands [9]. Interestingly, many co-occurring species significantly differ in mouthpart sizes and shapes, presumably owing to adaptations to a specialized diet [6,7], suggesting that stenophagy has evolved multiple times independently in these islands [10]. Although behavioral and physiological experiments have revealed a close correlation between morphological traits and prey preference in *Dysdera*, little is known about the molecular basis of trophic adaptations in this genus.

**Figure 1:**
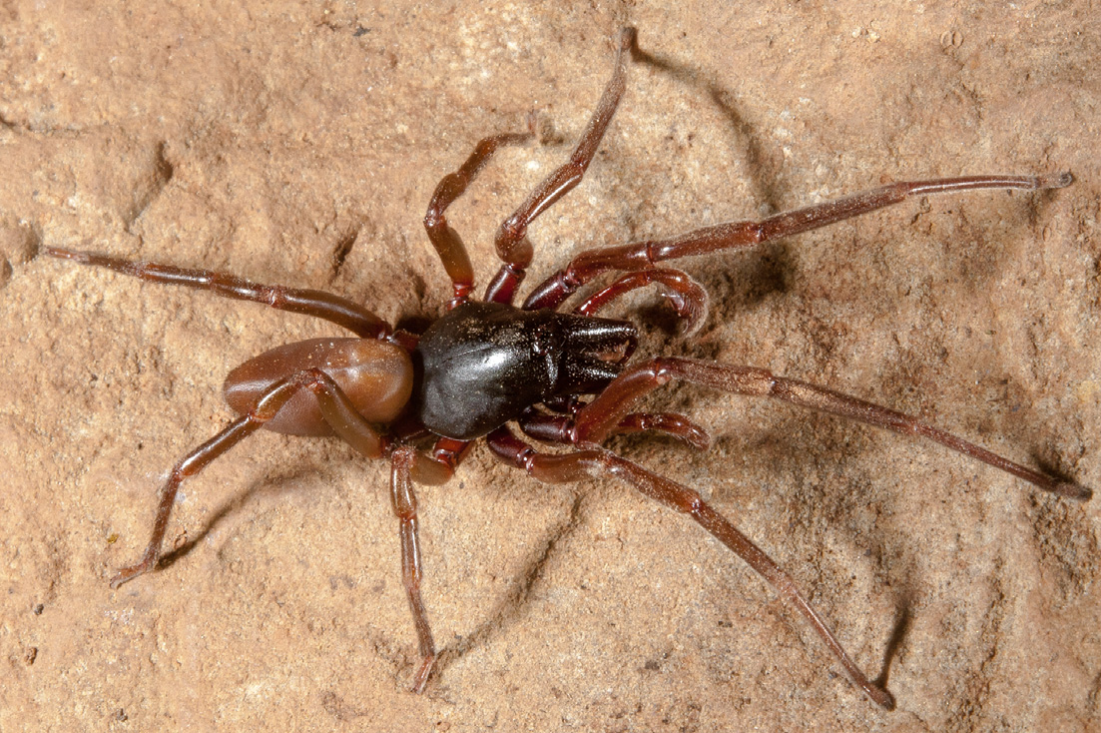
Male of *Dysdera silvatica* from Teselinde (La Gomera, Canary Islands). Photo credit: Miquel Arnedo.

**Figure 2:**
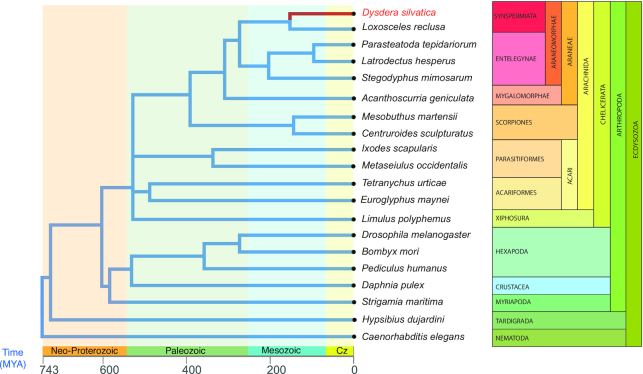
Phylogenetic relationships of the species used for the *D. silvatica* genome annotation (see Supplementary Table S1-11 for further details) and completeness analysis. Because the chelicerata phylogeny is controversial (e.g., [20], [21]), we set the most conflictive clades as polytomies. Divergence times were obtained from Carlson et al. (2017) [22] and the TimeTree web server (http://www.timetree.org/). Cz, cretaceous period.

Here we present the draft assembly and functional annotation of the genome of the Canary Island endemic spider *Dysdera silvatica* Schmidt, 1981 (NCBI:txid477319; Fig. 1). This study is the first genomic initiative within its family and just the second within the Synspermiata [11], a clade that includes most of the families formerly included in Haplogynae, which was recently shown to be paraphyletic [12,13] (Fig. 2). Remarkably, a recent review on arachnid genomics identified the superfamily Dysderoidea (namely, Dysderidae, Orsolobidae, Oonopidae, and Segestriidae) as one of the priority candidates for genome sequencing [14]. The new genome, intended to be a reference genome for genomic studies on trophic specialization, will also be a valuable source for the ongoing studies on the molecular components of the chemosensory system in chelicerates [15]. Besides, because of the numerous instances of independent adaptation to caves [16], the peculiar holocentric chromosomes [17], and the evidence for cryptic female choice mechanisms [18,19] within the family, the new genome will be a useful reference for the study of the molecular basis of adaptation to extreme environments, karyotype evolution, and sexual selection. Additionally, a new fully annotated spider genome will greatly improve our understanding of key features, such as the venom and silk. The availability of new genomic information in a sparsely sampled section of the tree of life of spiders [14] will further provide valuable knowledge about relevant scientific questions, such as gene content evolution across main arthropod groups, including the consequences of whole-genome duplications, or the phylogenetic relationships with Araneae.

## Sampling and DNA extraction

We sampled adult individuals of *D. silvatica* in different localities of La Gomera (Canary Islands) in March 2012 and June 2013 (Supplementary Table S1-1). The species was confirmed in the laboratory, and samples were stored at −80ºC until its use. For Illumina and PaciBio libraries (see below), we extracted genomic DNA using Qiagen DNeasy Blood & Tissue Kit (Qiagen, Hilden, Germany, 74104) ) according to the manufacturer’s protocol. For the Oxford Nanopore libraries, we used a modified version of the Blood & Cell Culture DNA Mini Kit (Qiagen). Due to the high amount of chitin present in spiders we incubated fresh original samples 48 h at 32ºC, avoiding a centrifugation step prior to sample loading to Qiagen Genomic tips, permitting the solution to precipitate by gravity. We also added an extra wash with 70% ethanol and centrifuged the solution at >5,000*g* for 10 min at 4ºC. We quantified the genomic DNA in a Qubit fluorometer (Life Technologies, Thermo Fisher Scientific Inc., USA) using the dsDNA BR (double stranded DNA Broad Range) Assay Kit and checked its purity in a NanoDrop 2000 spectrophotometer (Thermo Fisher Scientific Inc.).

## DNA sequencing

We sequenced the genome of *D. silvatica* using 4 different sequencing platforms (Table 1; Supplementary Table S1-2). First, we used the Illumina HiSeq2000 to obtain the genome sequence of a single male (100 bp, paired-end [PE] reads, 100 PE; TruSeq library). The flow-cell lane generated ∼51 Gb of sequence, representing a genome coverage of 30× (assuming a genome size of ∼1.7 Gb; see below). The genome of a female was sequenced using a mate pair (MP) approach; for that we used Nextera 5 kb-insert 100 PE libraries and the HiSeq2000 to generate ∼40 Gb of sequence (∼23× of coverage). A third individual (male) was used for single-molecule real-time (SMRT) sequencing (PacBio long reads). We used 8 SMRT libraries (20 kb SMRT bell templates), which were sequenced using the P6-C4 chemistry in a PacBio RSII platform. We obtained a yield of ∼9.6 Gb (raw coverage of ∼6×). Finally, 2 additional females were used for the 5 runs of Nanopore sequencing (Nanopore 1D libraries). We got a yield of ∼23.2 Gb (∼14× coverage) (Table 1; Supplementary Table S1-2).

**Table 1.** Sequencing data and library information

Run ID	Library	Insert size	Read lengths	Lanes	Total bases	Raw read pairs	Coverage (×)^a^
PE	Illumina HiSeq200 - Truseq	370 bp	100×100 PE	1	51,202,445,102	506,954,902	30
MP	Illumina HiSeq200 - Nextera	5 kb	100×100 PE	1	39,609,522,995	392,173,495	23
Nanopore	Nanopore 1D Libraries	-	Nanopore	5	23,193,357,481	20,534,058	14
PacBio	PacBio RSII 20 Kb SMRTbell	-	SMRT	8	9,652,844,880	1,455,288	6

^a^Based on the genome size estimated by flow cytometry ∼1.7 Gb.

## *D. silvatica* chromosome and genome size

*D. silvatica* has a diploid chromosome set of 6 pairs of autosomes and 2 (females are XX; 2n = 14) or 1 (males are X0) sex chromosomes (M. A. Arnedo, unpublished results). Using flow cytometry and the genome of the German cockroach *Blattella germanica* (1C = 2.025 Gb, J. S. Johnston, personal communication; see also [23]) as reference, we determined that the haploid genome size of *D. silvatica* is ∼1.7 Gb. For the analysis, we adapted the Hare and Johnston [24] protocol for spiders species, without using male palps and chelicers to avoid analyzing haploid or endoreplicated cells, respectively [25,26]. Shortly, we isolated cells from the head of the male cockroach, and legs and palps from female spiders. We incubated the cells in LB0.1 with 2% of tween [27], propidium iodide (50 μg/mL), and RNAse (40 μg/mL). After 10 minutes, the processed tissue was filtered using a nylon mesh of 20 μm. We determined the DNA content of the diploid cells through the relative G0/G1 peak positions of the stained nuclei using a Gallios flow cytometer (Beckman Coulter, Inc, Fullerton, CA); the results were based on the average of 3 spider replicates, counting a minimum of 5,000 cells per individual.

In addition, we also estimated the *D. silvatica* genome size from the distribution of *k*-mers (from short reads) with Jellyfish v.2.2.3 (Jellyfish, RRID:SCR_005491) [28]. The distribution of *k*-mers of size 17, 21, and 41 (GenomeScope (GenomeScope, RRID:SCR_017014) [29]) resulted in a haploid genome size of ∼1.23 Gb (Supplementary Fig. S1). The discrepancy between *k*-mer– and cytometry-based estimates may be caused by the presence of repetitive elements [30], which can affect *k*-mer estimates.

## Read preprocessing

To avoid including contaminants in the assembly step, we searched the raw reads for mitochondrial, bacterial, archaeal, and virus sequences. We downloaded all genomes of all these kinds available in the GenBank database (Supplementary Table S1-3) and used BLASTN v2.4.0 (BLASTN, RRID:SCR_001598) [31] to detect and filter all contaminant reads (E-value <10^−5^; >90% alignment length; >90% identity). We preprocessed raw reads using PRINSEQ v.0.20.3 (PRINSEQ, RRID:SCR_005454) [32]. We estimated some descriptive statistics, such as read length and *k*-mer representation, and calculated the amount of adapter sequences and exact duplicates.

Quality-based trimming and filtering was performed according to the chemistry, technology, and library used (Supplementary Table S1-4). For the short-insert 100 PE library, we used Trimmomatic v0.36 (Trimmomatic, RRID:SCR_011848) [33] with specific lists of adapters of the TruSeq v3 libraries to filter all reads shorter than 36 bp or with minimum quality scores < 30 along 4-bp sliding windows. We also filtered trailing and leading bases with a quality score < 10. Long-insert MP libraries were preprocessed using NxTrim v0.4.1 [34] with default parameters (Supplementary Table S1-4a and b). We preprocessed the raw PacBio reads using the SMRT Analysis Software (SMRT Analysis Software, RRID:SCR_002942) [35], by generating circularized consensus sequence to further perform a polishing analysis with Pilon v1.22 (Pilon, RRID:SCR_014731) [36] based on short reads (Supplementary Table S1-4c).

## *De novo* genome assembly

We used MaSuRCA v3.2.9 (MaSuRCA, RRID:SCR_010691) [37] for a hybrid *de novo* assembly of the *D. silvatica* genome (Supplementary Fig. S2). Additionally, we performed a scaffolding phase using AGOUTI (minimum number of joining reads pairs support, *k* = 3) [38], and the raw reads from a *D. silvatica* RNA sequencing (RNAseq) experiment [39] (Supplementary Table S1-5 and S1-6). During the assembly phase, we chose for each software the parameter values that generated the best assembly (Supplementary Table S1-7) in terms of (i) continuity and contig size statistics, such as the N50, L50, and the total number of sequences and bases assembled; and (ii) completeness measures, obtained as the fraction (and length) of a series of highly conserved proteins present in the draft genome. Particularly, we used 5 datasets, BUSCO v3 (BUSCO, RRID:SCR_015008) with genome option [40] using (i) the Arthropoda or (ii) the Metazoa dataset, (iii) the 457 core eukaryotic genes (CEGs) of *Drosophila melanogaster* [41], (iv) the 58,966 transcripts in the *D. silvatica* transcriptome [39], and (v) the 9,473 1:1 orthologs across 5 *Dysdera* species, *D. silvatica; D. gomerensis* Strand, 1911; *D. verneaui* Simon, 1883; *D. tilosensis* Wunderlich, 1992; and *D. bandamae* Schmidt, 1973 obtained from the comparative transcriptomics analysis of these species [42]. Finally, we performed an additional search to identify and remove possible contaminants in the generated scaffolds (Supplementary Table S1-7). We discarded 16 contaminant sequences > 5 kb. The final assembly size of the *D. silvatica* genome (Dsil v1.2) was ∼1.36 Gb, with an N50 of ∼38 kb (Table 2).

**Table 2.** *Dysdera silvatica* nuclear genome assembly and annotation statistics

Genome assembly^a^	Value
Assembly size (bp)	1,359,336,805
% AT/CG/N	64.91%/34.83%/0.26%
Number of scaffolds	65,205
Longest scaffold	340,047
N50	38,017
L50	10,436
Repeat statistics^b^	
Number of elements	3,284,969
Length (bp) [% Genome]	731,540,381 [53.81%]
Genome annotation^a^	
Protein-coding genes	48,619
Functionally annotated	36,398 (74.86%)
Without functional annotation	12,221 (25.14%)
tRNA genes	33,934

^a^See also Supplementary S1-7.

^b^Summary of the RepeatMasker analysis (See also Supplementary Table S1-9).

We determined the average genome coverage for each sequencing library with SAMtools v1.3.1 (SAMtools, RRID:SCR_002105) [43], by mapping short reads (using bowtie2 v2.2.9 [bowtie2, RRID:SCR_005476] [44]) or long reads (using minimap2 [45]) to the final draft assembly (Table 1; Supplementary Table S1-8; Supplementary Fig. S3).

## Repetitive DNA sequences

We analyzed the distribution of repetitive sequences in the genome of *D. silvatica*, using either a *de novo* with RepeatModeler v1.0.11 (RepeatModeler, RRID:SCR_015027) [46], or a database-guided search strategy with RepeatMasker v.4.0.7 (RepeatMasker, RRID:SCR_012954) [47]. We used 3 different databases of repetitive sequences, (i) *D. silvatica*–specific repetitive elements generated with RepeatModeler v1.0.11 [46], (ii) the Dfam_Consensus [48] (version 20170127), and (ii) the RepBase (version 20170127) [49,50]. We identified 2,604 families of repetitive elements, where 1,629 of them (62.6%) were completely unknown. Repetitive sequences accounted for ∼732 Mb, which represent 53.8% of the total assembly size (Table 2; Supplementary Table S1-9a). Remarkably, most abundant repeats are from unknown families, 22.6% of the assembled genome. The repetitive fraction of the genome also include DNA elements (16.8%), LINEs (10.7%), and SINEs (1.85%), and a small fraction of other elements, including LTR elements, satellites, simple repeats, and low-complexity sequences. We found that the 10 most abundant repeat families among the 2,604 identified in *D. silvatica* account for ∼7% of the genome and encode 5 unknown, 3 SINEs, and 2 LINEs, with an average length of ∼193, ∼161, and ∼1,040 bp, respectively (Supplementary Table S1-9b).

We also studied the distribution of the high-covered genome regions to describe the spacing pattern among repetitive sequences. In particular, we searched for genomic regions that have a higher than average sequencing coverage above a particular threshold. Because repetitive regions are more prone to form chimeric contigs in the assembly step, we only used MaSuRCA super reads, and longer than 10 kb and free of Ns (34,937 contigs; 1.12 Gb). We estimated the coverage after mapping the short reads (from the 100PE library) to those contigs. We defined as high-coverage regions (HCRs) those with a coverage ≥2.5× or 5× the genome-wide average (∼30×), in a region of ≥150, ≥500, ≥1,000, or ≥5,000 bp (Supplementary Fig. S4a; Supplementary Table S2). We found a large number of contigs encompassing ≥1 HCR. For instance, 21,614 contigs (∼61.9%) include ≥1 HCR of 150 bp with >2.5× coverage (an average of 2.48 HCRs per contig; 77.7 HCR per Mb) (Supplementary Table S2-2a). For HCRs of >5× coverage, the results are also remarkable (10,604 contigs have ≥1 HCR of 150 bp, corresponding to 25.6 HCR per Mb). As expected, the longer the HCR the smaller the fraction in the genome; indeed, we found that the genome is encompassing ∼5 HCR per Mb (HCR, longer than 1 kb at 2.5×). The distances between consecutive HCRs doenot show clear differences between the 2.5× and 5× thresholds (Supplementary Fig. 4b and S5; Supplementary Table S2-2b).

**Figure 3:**
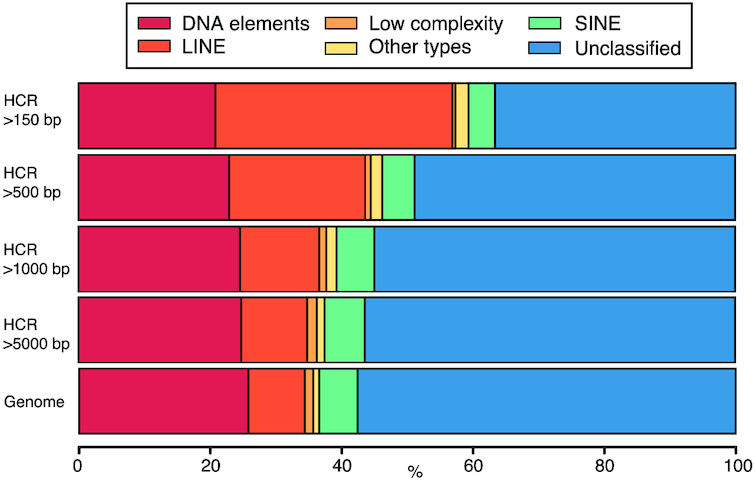
Bar plot of the annotation of the repetitive elements within the HCRs (2.5× threshold) at different intra-HCR length cutoffs (150, 500, 1,000, and 5,000 bp) (Supplementary Table S2-2a). Colors represent the type of repeat element identified by RepeatMasker. "Other types" class includes the LTR elements, small RNA, and satellite information that represent a small fraction.

We found a strong relationship between the length of the HCR and the type of the included repetitive elements (Fig. 3; Supplementary Table S2-3). For instance, while LINEs represent 8.62% of the repetitive elements in the whole genome, they are clearly enriched in the HCRs (36.12% in HCRs longer than 150 bp; 12.08% in HCRs longer than 5,000 bp) (Fig. 3; Supplementary Table S2-3a); the same was found for the small RNA fraction (ribosomal RNA). In contrast, the fraction of low-complexity repetitive sequences is much less represented in small HCRs than in the whole genome (∼1.3%). We also found that the coverage threshold has little effect on the results (Supplementary Table S2-3; Supplementary Fig. S6), either for the main families or across subfamilies (Supplementary Table S2-4 and S2-5).

Given that the HCR analysis covers an important fraction of the assembled bases (∼82%), the present results can likely be extrapolated to the whole genome. Therefore, the relatively low N50 of the *D. silvatica* genome draft is very likely to be caused by abundant interspersed repeats preventing genome continuity. Despite the low N50 we estimated that the draft presented here is mostly complete in terms of functional regions (see below).

## Transcriptome assembly and genome annotation

We used the newly generated genome sequence to obtain a reference-guided assembly of the *D. silvatica* transcriptome with the RNAseq data from Vizueta et al. [39]. We used HISAT2 v2.1.0 (HISAT2, RRID:SCR_015530) [51] to map the RNAseq reads to the reference and Trinity v2.4.0. (Trinity, RRID:SCR_013048) [52] (genome-guided bam, max intron = 50 kb, min coverage = 3) to assemble the transcriptome (named "Dsil-RefGuided transcriptome"; Supplementary Table S1-10). We used the MAKER2 v2.31.9 (MAKER2, RRID:SCR_005309) [53] genome annotation pipeline for the structural annotation of *D. silvatica* genes (Supplementary Fig. S2), using both *ab initio* gene predictions and annotation evidences from *D. silvatica* and other sources. For the *ab initio* gene predictions we initially trained Augustus v3.1.0 (Augustus, RRID:SCR_008417) [54] and SNAP (SNAP, RRID:SCR_002127) [55] softwares using scaffolds longer than 20 kb, and BUSCO gene models generated from completeness searches. Then we iteratively included a reliable set of proteins for a further training. This dataset was composed of the 9,473 orthologs 1:1 identified in 5 *Dysdera* species and the 1:1 orthologs among spiders available at OrthoDB v10 (OrthoDB, RRID:SCR_011980) [56] (8,792). After several iterative training rounds, we applied MAKER2, Augustus, and SNAP, adding other sources of evidence: (i) transcript evidence (Dsil-RefGuided transcriptome), (ii) RNAseq reads exon junctions generated with HISAT2 [51] and regtools [57], and (iii) proteins annotated in other arthropods, especially chelicerates (Fig. 2; Supplementary Table S1-11). The annotation process resulted in 48,619 protein-coding and 33,934 transfer RNA (tRNA) genes. The mean annotation edit distance (AED) upon protein-coding genes was 0.32 (Supplementary Fig. S6), which is typical of a well-annotated genome [58, 59]. After each training and iterative annotation round, we checked the improvement of the annotation by means of the cumulative fraction of AED (Supplementary Table S1-12a; Supplementary Fig. S7).

We searched for the presence of protein domain signatures in annotated protein-coding genes using InterProScan v5.15-54 (InterProScan, RRID:SCR_005829) [60,61], which includes information from public databases (see additional details in Supplementary Table S1-7). Additionally, we used NCBI BLASTP v.2.4.0 (BLASTP, RRID:SCR_001010) [31] (E-value cutoff <10^−5^; >75% alignment length) against the Swiss-Prot database to annotate *D. silvatica* genes. We found that 74.9% (36,398 genes) of the predicted protein-coding genes have hits with records of either InterPro (32,322 genes) (InterPro, RRID:SCR_006695) or Swiss-Prot (17,225 cases) (Table 2; Supplementary Table S1-7).

## Completeness

We determined the completeness of the *D. silvatica* genome assembly (Table 3) using BLASTP (E-value cutoff <10^−^^3^; >30% of alignment length and identity > 50%). We searched for homologs of the functionally annotated peptides (36,398) (i) among CEG genes of *Drosophila melanogaster* [41]; (ii) among the predicted peptides of *Parasteatoda tepidariorum*, a spider with a well-annotated genome [62]; (iii) among the 9,473 1:1 orthologs across 5 *Dysdera* species; and (iv) among the 2,198 single-copy genes identified in all spiders and available in OrthoDB v10 [56]. We found in *D. silvatica* a high fraction of putative homologs (95.8% of CEG genes, and 97.4% spider-specific single-copy genes; Table 3). Furthermore, the analysis based on the putative homologs of the single-copy genes included in the BUSCO dataset (BUSCO, RRID:SCR_015008) [40], applying the default parameters for the genome and protein mode, also demonstrated the high completeness of the genome draft. Indeed the analysis recovered the ∼90% of Metazoa or Arthropoda genes (v9), and nearly 70% of them are complete in *D. silvatica*.

**Table 3.** Completeness analysis^a^

**BLAST analysis^b^**	Number Identified (%)
Parasteatoda genes (*n* = 30,041)	19,580 (65.2)
Single-copy *Dysdera* (*n* = 9,473)	8,420 (88.9)
Single-copy spiders (*n* = 2,198)	2,141 (97.4)
CEG (*n* = 457)	438 (95.8)
**BUSCO analysis^c^**	
Metazoa (*n* = 978)	
Identified BUSCO	882 (90.2)
Complete (C)	689 (70.5)
Single copy (S)	662 (67.7)
Duplicated (D)	27 (2.8)
Fragmented (F)	193 (19.7)
Missing (M)	96 (9.8)
Artrhopoda (***n*= 1,066)**	
Identified BUSCO	959 (89.9)
Complete (C)	736 (69.1)
Single copy (S)	702 (65.9)
Duplicated (D)	34 (3.2)
Fragmented (F)	223 (20.9)
Missing (M)	107 (10.0)

^a^Completeness analysis of the 36,398 functional annotated proteins of *D. silvatica*.

^b^BLASTP searches against different datasets. E-value cutoff < 10^^−^3^, alignment length cutoff > 30%, and identity cutoff > 30%.

^c^BUSCO analysis using default parameters against different datasets (BUSCO, RRID:SCR_015008).

**Figure 4:**
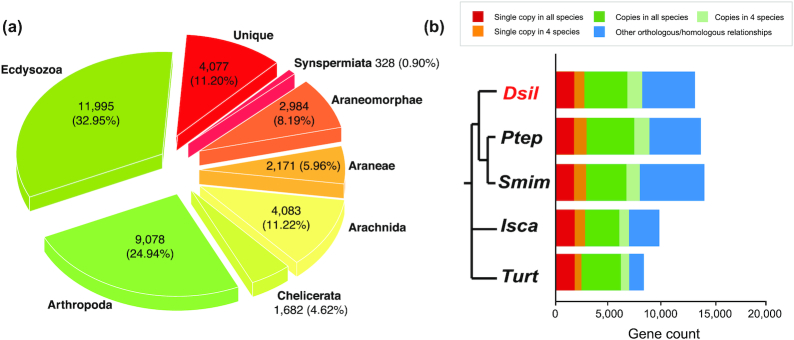
**(a)** Pie chart illustrating the taxonomic distribution of positive BLAST hits of the *D. silvatica* protein-coding genes against the sequence data of species included in Fig. 2. **(b)** Homology relationships among *D. silvatica* (Dsil) and different chelicerates genomes available in OrthoDB v10 [56], *Parasteatoda tepidariorum* (Ptep), *Stegodyphus mimosarum* (Smim), *Ixodes scapularis* (Isca), and *Tetranychus urticae* (Turt). Red and orange bars indicate the fraction of single-copy genes (1:1 orthologs) identified in all species, and in all but 1 (e.g., missing in 1 species), respectively. The dark and light green bar indicate the fraction of orthologs present in all species and in all but 1, respectively, that are not included in previous categories. The blue bar (other orthology/homology) shows other more complex homologous relationships. The results were generated by uploading *D. silvatica* proteins to the OrthoDB web server.

We extended the search for *D. silvatica* homologs to a broader taxonomic range (Fig. 2; Supplementary Table S1-11) by including other metazoan lineages and performing a series of local BLASTP searches (E-value cutoff < 10^^−^^^3^; >30% alignment length). We found that a great majority of *D. silvatica* genes are shared among arthropods (57.9%), 11,995 of them (32.95%) also being present in Ecdysozoa (Fig. 4a). Remarkably, 9,560 genes appears to be spider-specific, 4,077 of them being specific (unique) of *D. silvatica*. Despite almost all these species-specific genes having interproscan signatures, the annotation metrics are poor compared with genes having homologs in other species (Supplementary Table S1-12b; Supplementary Figs S7 and S9); indeed, they have an average number of exons (2.8) and gene length (∼168aa), which may reflect their partial nature. They could be part of very large genes interspersed by repeats or complex sequences difficult to assemble. The analysis using OrthoDB (v10) [56] across 5 chelicerates (including *D. silvatica*) identified 1,798 genes, with 1:1 orthologous relationships (Fig. 4b), while 12,101 *D. silvatica* genes showed other more complex orthologous/homologous relationships (Fig. 4b, Supplementary Table S1-12c and S3-1). The analysis across the genome annotations of some representative arthropods identified 950 genes with 1:1 orthologous relationships (Supplementary Fig. S8, Supplementary Table S1-12c and S3-2).

## Mitochondrial genome assembly and annotation

We assembled the mitochondrial genome of *D. silvatica* (mtDsil) from 126,758 reads identified in the 100PE library by the software NOVOPlasty [63]. Our *de novo* assembly yielded a unique contig of 14,440 bp (coverage of 878×) (Supplementary Table S1-13). CGVIEW (CGVIEW, RRID:SCR_011779) [64] was used to generate a genome visualization of the annotated mtDsil genome (Supplementary Fig. S10). We identified 2 ribosomal RNAs, 13 protein-coding genes, and 15 tRNAs (out of the putative 22 tRNAs). Based on the contig length and the inability of standard automatic annotation algorithms to identify tRNA with missing arms, as reported for spiders [65], the complete set of tRNAs is most likely present for this species.

## Conclusion

We have reported the assembly and annotation of the nuclear and mitochondrial genomes of the first representative of the spider superfamily Dysderoidea and the second genome of a Synspermiata, one of the main evolutionary lineages within the “true spiders” (Araneomorphae) and still sparsely sampled at the genomic level [14]. Despite the high coverage and the hybrid assembly strategy, the repetitive nature of the *D. silvatica* genome precluded obtaining a high-continuity draft. The characteristic holocentric chromosomes of Dysderidae [17] may also explain the observed genome fragmentation; indeed, it has been recently shown that genome-wide centromere-specific repeat arrays are interspersed among euchromatin in holocentric plants (Rhynchospora, Cyperceae) [66].

Nevertheless, the completeness and the extensive annotations achieved for this genome, as well as the new reference-guided transcriptome, make this draft an excellent source tool for further functional and evolutionary analyses in this and other related species, including the origin and evolution of relevant spider traits, such as venom and silk. Moreover, the availability of new genomic information in a lineage with remarkable evolutionary features such as recurrent colonizations of the underground environment or complex reproductive anatomies indicative of cryptic female choice, to cite 2 examples, will further provide valuable knowledge about relevant scientific questions, such as the molecular basis of adaptation to extreme habitats or the genetic drivers of sexual selection, along with more general aspects related to gene content across main arthropod groups, the consequences of whole-genome duplications, or phylogenetic relationships with the Araneae. Additionally, because this genus experienced a spectacular adaptive radiation in the Canary Islands, the present genome draft could be useful to further studies investigating the genomic basis of island radiations.

## Availability of supporting data and materials

The whole-genome shotgun project has been deposited at DDBJ/ENA/GenBank under accession number QLNU00000000 and project ID PRJNA475203. The version described in this article is version QLNU01000000. This project repository includes raw data, sequencing libraries information, and assemblies of the mitochondrial and nuclear genomes. Other relevant datasets such as annotation, reference-guide assembled transcripts, repeat, and HCR data, as well as other data relevant for the reproducibility of results, are available in the GigaDB dataset [67].

## Additional file

**File S1. Supplemental Material Summary**

**SanchezHerrero_Dsilvatica_SupMaterial_Summary.pdf**

## Availability of supporting source code and requirements

The scripts employed and developed in this project are available under the github repository:

Project name: Genome assembly of *Dysdera silvatica*

Project home page: https://github.com/molevol-ub/Dysdera_silvatica_genome

Operating system(s): Platform independent

Programming language: Bash, Perl, Python, R

License: MIT

## Abbreviations

AED: annotation edit distance; AGOUTI: Annotated Genome Optimization Using Transcriptome Information; BLAST: Basic Local Alignment Tool; bp: base pair; BUSCO: Benchmarking Universal Single Copy Orthologs; CEG: core eukaryotic gene; Cz: Cretaceous period; Dsil: *Dysdera silvatica*; Gb: gigabase pairs; GC: guanine cytosine; GO: Gene Ontology; HCR: high-coverage regions; Isca: *Ixodes scapularis*; kb: kilobase pairs; LINE: long interspersed nuclear element; LTR: long terminal repeats; MaSuRCA: Maryland Super-Read Celera Assembler; Mb: megabase pairs; MP: mate pair; Mya: million years ago; NCBI: National Center for Biotechnology Information; PacBio: Pacific Biosciences; PE: paired-end; PRINSEQ: PReprocessing and INformation of SEQuence data; Ptep: *Parasteatoda tepidariorum*; RNAseq: RNA sequencing; SINE: short interspersed nuclear element; Smim: *Stegodyphus mimosarum*; SMRT: Single-Molecule Real Time; tRNA: transfer RNA; Turt: *Tetranychus urticae*.

## Competing interests

The authors declare that they have no competing interests.

## Funding

This study was supported by the Ministerio de Economía y Competitividad of Spain (CGL2012-36863, CGL2013-45211, and CGL2016-75255), and by the Comissió Interdepartamental de Recerca I Innovació Tecnològica of Catalonia, Spain (2014SGR-1055 and 2014SGR1604). J.F.S.-H. was supported by a Formación del Profesor Universitario (FPU) grant (Ministerio de Educación of Spain, FPU13/0206); C.F.-L. by an IRBio PhD grant; S.H-A by Becas Postdoctorales en el Extranjero CONACyT; A.S.-G. by a Beatriu de Pinós grant (Generalitat de Catalunya, 2010-BP-B 00175); and J.R. and M.A.A. were partially supported by ICREA Academia (Generalitat de Catalunya).

## Authors' contributions

J.R., A.S.-G., and M.A.A designed the study. C.F.-L., J.F.S.-H., P.E., and S.H-A. processed the samples and extracted DNA. J.F.S.-H. performed the bioinformatics analysis and drafted the manuscript. J.F.S.-H., A.S.-G., and J.R. interpreted the data. All authors revised and approved the final manuscript.

## Supplementary Material

giz099_GIGA-D-19-00156_Original_Submission

giz099_GIGA-D-19-00156_Revision_1

giz099_GIGA-D-19-00156_Revision_2

giz099_GIGA-D-19-00156_Revision_3

giz099_GIGA-D-19-00156_Revision_4

giz099_Response_to_Reviewer_Comments_Original_Submission

giz099_Response_to_Reviewer_Comments_Revision_1

giz099_Response_to_Reviewer_Comments_Revision_2

giz099_Response_to_Reviewer_Comments_Revision_3

giz099_Reviewer_1_Report_Original_Submission Tatsuhiko Kadowaki -- 5/29/2019 Reviewed

giz099_Reviewer_2_Report_Original_Submission Nadia Ayoub -- 6/11/2019 Reviewed

giz099_Supplemental_Files
