# Supplementary material for: The draft genome sequence of the spider Dysdera silvatica (Araneae, Dysderidae): A valuable resource for functional and evolutionary genomic studies in chelicerates
Source: Gigascience. 2019 Aug 20;8(8):giz099. doi: 10.1093/gigascience/giz099 (PMC6701490; doi:10.1093/gigascience/giz099)
Supplement: giz099_GIGA-D-19-00156_Revision_4 [file giz099_giga-d-19-00156_revision_4.pdf]

GigaScience

The draft genome sequence of the spider Dysdera silvatica (Araneae, Dysderidae): A valuable resource for functional and evolutionary genomic studies in chelicerates

--Manuscript Draft--

**Manuscript Number:**	GIGA-D-19-00156R4
**Full Title:**	The draft genome sequence of the spider Dysdera silvatica (Araneae, Dysderidae): A valuable resource for functional and evolutionary genomic studies in chelicerates
**Article Type:**	Data Note
**Funding Information:**	Ministerio de Economía, Industria y Competitividad, Spain (CGL2012-36863) — Prof. Miquel A. Arnedo<br>Ministerio de Economía, Industria y Competitividad, Spain (CGL2013-45211) — Prof. Julio Rozas<br>Ministerio de Economía, Industria y Competitividad, Spain (CGL2016-75255) — Prof. Julio Rozas<br>Comissió Interdepartamental de Recerca i Innovació Tecnològica, Generalitat de Catalunya, Spain (2014SGR-1055) — Prof. Julio Rozas<br>Comissió Interdepartamental de Recerca i Innovació Tecnològica, Generalitat de Catalunya, Spain (2014SGR-1604) — Prof. Miquel A. Arnedo<br>Comissió Interdepartamental de Recerca i Innovació Tecnològica, Generalitat de Catalunya, Spain (2017 SGR 1287) — Prof. Julio Rozas<br>Comissió Interdepartamental de Recerca i Innovació Tecnològica, Generalitat de Catalunya, Spain (2017 SGR 83) — Prof. Miquel A. Arnedo
**Abstract:**	We present the draft genome sequence of Dysdera silvatica, a nocturnal ground dwelling spider from a genus that undergone a remarkable adaptive radiation in the Canary Islands. The draft assembly was obtained using short (illumina) and long (PacBio and Nanopore) sequencing reads. Our de novo assembly (1.36 Gb), that represents the 80% of the genome size estimated by flow-cytometry (1.7 Gb), is constituted by a high fraction of interspersed repetitive elements (53.8%). The assembly completeness, using BUSCO (benchmarking universal single-copy orthologs) and CEG (Core Eukaryotic genes), ranges from 90% to 96%. Functional annotations based on both ab initio and evidence-based information (including D. silvatica RNA-seq) yielded a total of 48 619 protein-coding sequences, of which 36 398 (74.9%) have the molecular hallmark of known protein domains, or sequence similarity with swissprot sequences. The D. silvatica assembly is the first representative of the superfamily Dysderoidea, and just the second available genome of Synspermiata, one of the major evolutionary lineages of the “true spiders” (Araneomorphae). Dysderoids, which are known for their numerous instances of adaptation to underground environments, include some of the few examples of trophic specialization within spiders and are excellent models for the study of cryptic female choice. This resource, will be therefore very useful as an starting point to study fundamental evolutionary and functional questions, including the molecular bases of the adaptation to extreme environments and ecological shifts, as well of the origin and evolution of relevant spider traits, such as the venom and silk.
**Corresponding Author:**	Julio Rozas, PhD in Biology<br>Universitat de Barcelona<br>Barcelona, SPAIN
**Corresponding Author Secondary Information:**	

Powered by Editorial Manager® and ProduXion Manager® from Aries Systems Corporation

Corresponding Author's Institution:	Universitat de Barcelona
Corresponding Author's Secondary Institution:	
First Author:	Julio Rozas, PhD in Biology
First Author Secondary Information:	
Order of Authors:	Julio Rozas, PhD in Biology
	Sánchez-Herrero Jose Francisco, Master in Bioinformatics
	Cristina Frías-López, Master in Genetics
	Paula Escuer, Master in Genetics
	Silvia Hinojosa-Alvarez, PhD in Biology
	Miquel A. Arnedo, PhD in Biology
	Alejandro Sánchez-Gracia, PhD in Biology
Order of Authors Secondary Information:	
Response to Reviewers:	I am sending the revised PDF, including your suggested changes.
Additional Information:	
**Question**	**Response**
Are you submitting this manuscript to a special series or article collection?	No
**Experimental design and statistics**<br><br>Full details of the experimental design and statistical methods used should be given in the Methods section, as detailed in our Minimum Standards Reporting Checklist. Information essential to interpreting the data presented should be made available in the figure legends.<br><br>Have you included all the information requested in your manuscript?	Yes
**Resources**<br><br>A description of all resources used, including antibodies, cell lines, animals and software tools, with enough information to allow them to be uniquely identified, should be included in the Methods section. Authors are strongly encouraged to cite Research Resource Identifiers (RRIDs) for antibodies, model organisms and tools, where possible.	Yes

Powered by Editorial Manager® and ProduXion Manager® from Aries Systems Corporation

Have you included the information requested as detailed in our Minimum Standards Reporting Checklist?	
**Availability of data and materials**<br><br>All datasets and code on which the conclusions of the paper rely must be either included in your submission or deposited in publicly available repositories (where available and ethically appropriate), referencing such data using a unique identifier in the references and in the “Availability of Data and Materials” section of your manuscript.<br><br>Have you have met the above requirement as detailed in our Minimum Standards Reporting Checklist?	Yes

Powered by Editorial Manager® and ProduXion Manager® from Aries Systems Corporation

Manuscript
Click here to access/download;Manuscript;20190726_Dsil_genome_paper.p
Click here to view linked References

OXFORD (GIGA)^n SCIENCE

GigaScience, 2019, 1–9

doi: xx.xxxx/xxxx
Manuscript in Preparation
DATA NOTE

DATA NOTE

The draft genome sequence of the spider *Dysdera silvatica* (Araneae, Dysderidae): A valuable resource for functional and evolutionary genomic studies in chelicerates

José Francisco Sánchez-Herrero[1,2], Cristina Frías-López[1,2], Paula Escuer[1,2], Silvia Hinojosa-Alvarez[1,2,3], Miquel A. Arnedo[2,4], Alejandro Sánchez-Gracia[1,2,*] and Julio Rozas[1,2,*]

[1]Departament de Genètica, Microbiologia i Estadística, Universitat de Barcelona (UB), Barcelona, Spain and [2]Institut de Recerca de la Biodiversitat (IRBio) (UB) and [3]Jardín Botánico, Instituto de Biología, Universidad Nacional Autónoma de México, Ciudad de México, México and [4]Departament de Biologia Evolutiva, Ecologia i Ciències Ambientals (UB)

[*]Correspondence address: Departament de Genètica, Microbiologia i Estadística, Facultat de Biologia, Avenida Diagonal 643, 08028, Barcelona, Spain. Alejandro Sánchez-Gracia (E-mail: elsanchez@ub.edu, https://orcid.org/0000-0003-4543-4577) and Julio Rozas (E-mail: jrozas@ub.edu, https://orcid.org/0000-0002-6839-9148)

Abstract

We present the draft genome sequence of *Dysdera silvatica*, a nocturnal ground dwelling spider from a genus that undergone a remarkable adaptive radiation in the Canary Islands. The draft assembly was obtained using short (illumina) and long (PacBio and Nanopore) sequencing reads. Our *de novo* assembly (1.36 Gb), that represents the 80% of the genome size estimated by flow-cytometry (1.7 Gb), is constituted by a high fraction of interspersed repetitive elements (53.8%). The assembly completeness, using BUSCO (benchmarking universal single-copy orthologs) and CEG (Core Eukaryotic genes), ranges from 90% to 96%. Functional annotations based on both *ab initio* and evidence-based information (including *D. silvatica* RNA-seq) yielded a total of 48,619 protein-coding sequences, of which 36,398 (74.9%) have the molecular hallmark of known protein domains, or sequence similarity with swissprot sequences. The *D. silvatica* assembly is the first representative of the superfamily Dysderoidea, and just the second available genome of Synspermiata, one of the major evolutionary lineages of the "true spiders" (Araneomorphae). Dysderoids, which are known for their numerous instances of adaptation to underground environments, include some of the few examples of trophic specialization within spiders and are excellent models for the study of cryptic female choice. This resource, will be therefore very useful as a starting point to study fundamental evolutionary and functional questions, including the molecular bases of the adaptation to extreme environments and ecological shifts, as well of the origin and evolution of relevant spider traits, such as the venom and silk.

Key words: Spiders, *De novo* genome assembly, genome annotation, *Dysdera silvatica*.

Data Description

Spiders are a highly diverse and abundant group of predatory arthropods, found in virtually all terrestrial ecosystems. Ap-

Compiled on: July 26, 2019.
Draft manuscript prepared by the author.

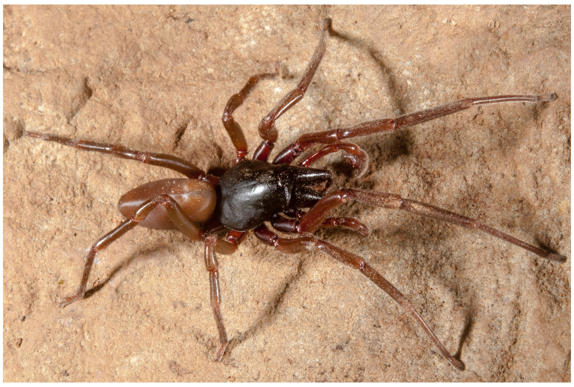

Figure 1. Male of *Dysdera silvatica* from Teselinde (La Gomera, Canary Islands). Photo credit Miquel Arnedo

proximately 45,000 spider species have been recorded to date [1]. The nocturnal ground family Dysderidae, ranks 17th out of 118 currently accepted spider families in number of species. The type genus of the family, *Dysdera* Latreille, 1804, includes half of the family diversity (282 species). This genus is remarkable in several aspects. First, it represents one of the few cases of stenophagy, i.e. prey specialization, across spiders [2]. Many species in the genus have evolved special morphological, behavioral and physiological adaptations to feed on woodlice, including modifications of mouthparts, unique hunting strategies and effective restriction to assimilation of metals into its tissues [3, 4, 5, 6, 7]. Because of their chemical defenses and ability to accumulate heavy metals from the soil, woodlice are usually avoided as prey by most spiders, including generalist *Dysdera* [2, 4, 5, 7]. Although mostly circumscribed to the Mediterranean region, *Dysdera* has colonized all the Macaronesian archipelagoes, and has undergone a remarkable species diversification in the Canary Islands [8]. As many as 55 species have been recorded across the seven main islands and islets of this archipelago, being most of them single-island endemics [9]. Although multiple colonization events may account for the initial origin of species diversity the bulk of this diversity is the result of *in situ* diversification [8]. *Dysdera* spiders have adapted to a broad range of terrestrial habitats within Canary Islands [9]. Interestingly, many co-occurring species significantly differ in mouthpart sizes and shapes, presumably due to adaptations to a specialized diet [6, 7], suggesting that stenophagy has evolved multiple times independently in these islands [10]. Although behavioral and physiological experiments have revealed a close correlation between morphological traits and prey preference in *Dysdera*, little is known about the molecular basis of trophic adaptations in this genus.

Here we present the draft assembly and functional annotation of the genome of the Canary Island endemic spider *Dysdera silvatica* Schmidt, 1981 (NCBI Taxon ID: 477319; Figure 1). This study is the first genomic initiative within its family and just the second within the Synspermiata [11], a clade that includes most of the families formerly included in Haplogynae, which was recently shown to be paraphyletic [12, 13] (Figure 2). Remarkably, a recent review on arachnid genomics identified the superfamily Dysderoidea (namely Dysderidae, Orsolobidae, Oonopidae and Segestriidae) as one of the priority candidates for genome sequencing [14]. The new genome, intended to be a reference genome for genomic studies on trophic specialisation, will also be a valuable source for the ongoing studies on the molecular components of the chemosensory system in chelicerates [15]. Besides, because of the numerous instances of independent adaptation to caves [16], the peculiar holocentric chromosomes [17], and the evidence for cryptic female choice mechanisms [18, 19] within the family, the new genome will be a useful reference for the study of the molecular basis of adaptation to extreme environments, karyotype evolution and sexual selection. Additionally, a new fully annotated spider genome will greatly improve our understanding of key features, such as the venom and silk. The availability of new genomic information in a sparsely sampled section of the tree of life of spiders [14] will further provide valuable knowledge about relevant scientific questions, such as gene content evolution across main arthropod groups, including the consequences of whole genome duplications, or the phylogenetic relationships with Araneae.

Sampling and DNA extraction

We sampled adult individuals of *D. silvatica* in different localities of La Gomera (Canary Islands) in March 2012 and June 2013 (Supplementary Table S1-1). The species was confirmed in the laboratory and samples were stored at -80ºC until its use. For Illumina and PacBio libraries (see below), we extracted genomic DNA (gDNA) using Qiagen DNeasy Blood & Tissue Kit (Qiagen, Germany) according to the manufacturer's protocol. For the Oxford Nanopore libraries, we used a modified version of the Blood & Cell Culture DNA Mini Kit (Qiagen, Germany). Due to the high amount of chitin present in spiders we incubated fresh original samples 48 h at 32ºC, avoiding a centrifugation step prior to sample loading to Qiagen Genomic tips permitting the solution to precipitate by gravity. We also added an extra wash with 70% Ethanol and centrifuged the solution at > 5,000 g for 10 min at 4ºC. We quantified the gDNA in a Qubit fluorometer (Life Technologies, Thermo Fisher Scientific) using the dsDNA BR (double stranded DNA Broad Range) Assay Kit, and checked its purity in a NanoDrop 2000 spectrophotometer (Thermo Fisher Scientific).

DNA sequencing

We obtained the genome of *D. silvatica* using four different sequencing platforms (Table 1; Supplementary Table S1-2). First, we used the Illumina HiSeq2000 to obtain the genome sequence of a single male (100 bp, paired-end reads, 100 PE; TruSeq library). The flow-cell lane generated about 51 Gb of sequence, representing a genome coverage of 30X (assuming a genome size of ~1.7 Gb; see below). The genome of a female was sequenced using a Mate Pair approach; for that we used Nextera 5 kb-insert 100 PE libraries and the HiSeq2000 to generate

Table 1. Sequencing data and library information.

Run ID	Library	Insert Size	Read Lengths	Lanes	Total Bases	Raw Read Pairs	Coverage[a]
PE	Illumina HiSeq200 - Truseq	370 bp	100x100 PE	1	51,202,445,102	506,954,902	30X
MP	Illumina HiSeq200 - Nextera	5 Kb	100x100 PE	1	39,609,522,995	392,173,495	23X
Nanopore	Nanopore 1D Libraries	-	Nanopore	5	23,193,357,481	20,534,058	14X
PacBio	PacBio RSII 20 Kb SMRTbell	-	SMRT	8	9,652,844,880	1,455,288	6X

[a]Based on the genome size estimated by flow cytometry ~1.7 Gb.

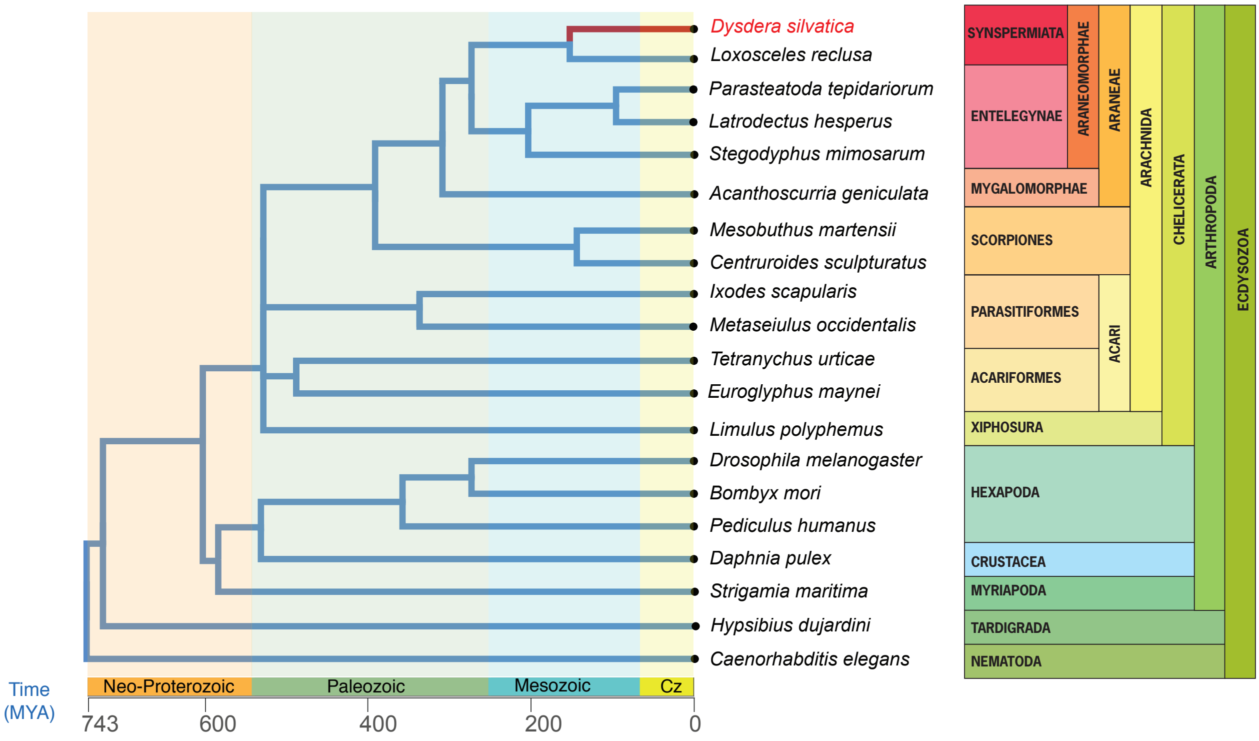

Figure 2. Phylogenetic relationships of the species used for the *D. silvatica* genome annotation (see Supplementary Table S1-11 for further details) and completeness analysis. Since the chelicerata phylogeny is very controversial (eg. [20], [21]), we set the most conflictive clades as polytomies. Divergence times were obtained from Carlson et al. (2017) [22] and the TimeTree web server (`http://www.timetree.org/`). Cz, cretaceous period.

about 40 Gb of sequence (about 23X of coverage). A third individual (male) was used for single-molecule real-time (SMRT) sequencing (PacBio long reads). We used 8 SMRT libraries (20 kb SMRT bell templates), which were sequenced using the P6-C4 chemistry in a PacBio RSII platform. We obtained a yield of about 9.6 Gb (raw coverage of about 6X). Finally, two additional females were used for the 5 runs of Nanopore sequencing (Nanopore 1D libraries). We got a yield of about 23.2 Gb (about 14X coverage) (Table 1; Supplementary Table S1-2).

D. silvatica chromosome and genome size

D. silvatica has a diploid chromosome set of 6 pairs of autosomes and two (females are XX; 2n = 14) or one (males are X0) sex chromosomes (M. A. Arnedo, unpublished results). Using flow cytometry and the genome of the German cockroach *Blattella germanica* (1C = 2.025 Gb, J. S. Johnston, personal communication; see also [23] as reference, we determined that the haploid genome size of *D. silvatica* is ~1.7 Gb. For the analysis, we adapted the Hare and Johnston [24] protocol for spiders species, without using male palps and chelicers to avoid analyzing haploid or endoreplicated cells, respectively [25, 26]. Shortly, we isolated cells from the head of male cockroach, and legs and palps from female spiders. We incubated the cells in LB0.1 with 2% of tween [27], Propidium Iodide (50 µg/mL) and RNAse (40 µg/mL). After 10 minutes, the processed tissue was filtered using a nylon mesh of 20 µm. We determined the DNA content of the diploid cells through the relative G0/G1 peak positions of the stained nuclei using a Gallios flow cytometer (Beckman Coulter, Inc, Fullerton, CA); the results were based on the average of 3 spider replicates, counting a minimum of 5,000 cells per individual.

In addition, we also estimated the *D. silvatica* genome size from the distribution of *k*-mers (from short reads) with Jellyfish v.2.2.3 (Jellyfish, RRID:SCR_005491) [28]. The distribution of *k*-mers of size 17, 21 and 41 (GenomeScope (GenomeScope, RRID:SCR_017014) [29]) resulted in an haploid genome size of about ~1.23 Gb (Supplementary Figure S1). The discrepancy between *k*-mer- and cytometry-based estimates may be caused to the presence of repetitive elements [30], which can affect *k*-mers estimates.

Read pre-processing

To avoid including contaminants in the assembly step, we searched the raw reads for mitochondrial, bacterial, archaeal and virus sequences. We downloaded all genomes of all these kinds available in the GenBank database (Supplementary Table S1-3), and used BLASTN v2.4.0 (BLASTN, RRID:SCR_001598) [31] to detect and filter all contaminant reads (E-value < 10^{-5}; > 90% alignment length; > 90% identity). We pre-processed raw reads using PRINSEQ v.0.20.3 (PRINSEQ, RRID:SCR_005454) [32]. We estimated some descriptive statistics, such as read-length and *k*-mer representation, and calculated the amount of adapter sequences and exact duplicates.

Quality-based trimming and filtering was performed according to the chemistry, technology and library used (Supplementary Table S1-4). For the short-insert 100 PE library, we used Trimmomatic v0.36 (Trimmomatic, RRID:SCR_011848) [33] with specific lists of adapters of the TruSeq v3 libraries to filter all reads shorter than 36bp or with minimum quality scores < 30 along a 4 bp sliding windows. We also filtered trailing and leading bases with a quality score < 10. Long-insert MP libraries were pre-processed using NxTrim v0.4.1 [34] with default parameters (Supplementary Table S1-4a & S1-4b). We pre-processed the raw PacBio reads using the SMRT Analysis Software (SMRT Analysis Software, RRID:SCR_002942) [35], by generating circularized consensus sequence (CCS) to further perform a polishing analysis with Pilon v1.22 (Pilon, RRID:SCR_014731) [36] based on short reads (Supplementary

Table S1-4c).

De novo genome assembly

We used MaSuRCA v3.2.9 (MaSuRCA, RRID:SCR_010691) [37] for an hybrid *de novo* assembly of the *D. silvatica* genome (Supplementary Figure S2). Additionally, we performed a scaffolding phase using AGOUTI (minimum number of joining reads pairs support, k = 3) [38], and the raw reads from a *D. silvatica* RNAseq experiment [39] (Supplementary Table S1-5 & S1-6). During the assembly phase, we chose for each software the parameter values that generated the best assembly (Supplementary Table S1-7) in terms of, i) continuity and contig size statistics, such as the N50, L50, and the total number of sequences and bases assembled, and ii) completeness measures, obtained as the fraction (and length) of a series of highly conserved proteins present in the draft genome. Particularly, we used five data sets, BUSCO v3 (BUSCO, RRID:SCR_015008) with genome option [40] using i) the Arthropoda or ii) the Metazoa dataset, iii) the 457 Core Eukaryotic Genes (CEG) of Drosophila melanogaster [41], iv) the 58,966 transcripts in the *D. silvatica* transcriptome [39] and, v) the 9,473 1:1 orthologs across five *Dysdera* species, *D. silvatica*, *D. gomerensis* Strand, 1911; *D. verneaui* Simon, 1883; *D. tilosensis* Wunderlich, 1992 and *D. bandamae* Schmidt, 1973 obtained from the comparative transcriptomics analysis of these species [J. Vizueta et al., unpublished results]. Finally, we performed an additional search to identify and remove possible contaminants in the generated scaffolds (Supplementary Table S1-7). We discarded 16 contaminant sequences > 5 kb. The final assembly size of the *D. silvatica* genome (Dsil v1.2) was ~1.36 Gb, with a N50 of about 38 kb (Table 2).

We determined the average genome coverage for each sequencing library with SAMtools v1.3.1 (SAMtools, RRID:SCR_002105) [42], by mapping short reads (using bowtie2 v2.2.9 (bowtie2, RRID:SCR_005476) [43]) or long reads (using minimap2 [44]) to the final draft assembly (Table 1; Supplementary Table S1-8; Supplementary Figure S3).

Repetitive DNA sequences

We analyzed the distribution of repetitive sequences in the genome of *D. silvatica*, using either a *de novo* with RepeatModeler v1.0.11 (RepeatModeler, RRID:SCR_015027) [45], and a database guided search strategy with RepeatMasker v.4.0.7 (RepeatMasker, RRID:SCR_012954) [46]. We used three different databases of repetitive sequences, i) *D. silvatica* specific repetitive elements generated with RepeatModeler v1.0.11 [45], ii) the Dfam_Consensus [47] (version 20170127), and ii) the RepBase (version 20170127) [48, 49]. We identified 2,604 families of repetitive elements, where 1,629 of them (62.6%) were completely unknown. Repetitive sequences accounted for ~732 Mb, which represent 53.8% of the total assembly size (Table 2; Supplementary Table S1-9a). Remarkably, most abundant repeats are from unknown families, 22.6% of the assembled genome. The repetitive fraction of the genome also include DNA elements (16.8%), LINEs (10.7%) and SINEs (1.85%), and a small fraction of other elements, including LTR elements, satellites, simple repeats and low complexity sequences. We found that the 10 most abundant repeat families among the 2,604 identified in *D. silvatica* account for ~7% of the genome and encode 5 unknown, 3 SINEs and 2 LINEs, with an average length of ~193 bp, ~161 bp and ~1040 bp, respectively (Supplementary Table S1-9b).

We also studied the distribution of the high covered genome regions to describe the spacing pattern among repetitive sequences. In particular, we searched for genomic regions that have a higher than average sequencing coverage above a particular threshold. Since repetitive regions are more prone to form chimeric contigs in the assembly step, we only used MaSuRCA super reads, and longer than 10 kb and free of Ns (34,937 contigs; 1.12 Gb). We estimated the coverage after mapping the short reads (from the 100PE library) to those contigs. We defined as high coverage regions (HCR) those with a coverage equal or greater than a 2.5 or 5 times the genome-wide average (~30X), in a region of at least (150, 500, 1000 or 5000 bp, Supplementary Figure S4a; Supplementary Table S2). We found a large number of contigs encompassing one or more HCR. For instance, 21,614 contigs (~61.9%) include at least one 150 bp HCR region with more than 2.5x coverage (an average of 2.48 HCRs per contig; 77.7 HCR per Mb) (Supplementary Table S2-2a). For HCRs of more than 5x coverage, the results are also remarkable (10,604 contigs have at least one 150 bp HCR, corresponding to 25.6 HCR per Mb). As expected, the longer the HCR the smaller the fraction in the genome; indeed, we found that the genome is encompassing ~5 HCR per Mb (HCR, longer than 1 kb at 2.5x). The distances between consecutive HCRs does not show clear differences between the 2.5x and 5x thresholds (Supplementary Figure 4b & S5; Supplementary Table S2-2b).

We found a strong relationship between the length of the HCR and the type of the included repetitive elements (Figure 3; Supplementary Table S2-3). For instance, while LINEs represent 8.62% of the repetitive elements in the whole genome, they are clearly enriched in the HCRs (36.12% in HCRs longer than 150 bp; 12.08% in HCRs longer than 5,000 bp) (Figure 3; Supplementary Table S2-3a); the same was found for the small RNA fraction (rRNA). In contrast, the fraction of low complexity repetitive sequences is much less represented in small HCRs than in the whole genome (about 1.3%). We also found that the coverage threshold has little effect on the results (Supplementary Table S2-3; Supplementary Figure S6), either for the main families or across subfamilies (Supplementary Table S2-4 & S2-5).

Given that the HCR analysis covers an important fraction of the assembled bases (~82%), current results can likely be extrapolated to the whole genome. Therefore, the relatively low N50 of the *D. silvatica* genome draft is very likely to be caused by

Table 2. *Dysdera silvatica* nuclear genome assembly and annotation statistics.

**Genome Assembly**[a]	
Assembly Size (bp)	1,359,336,805
% AT / CG / N	64.91% / 34.83% / 0.26%
Number of Scaffolds	65,205
Longest scaffold	340,047
N50	38,017
L50	10,436
**Repeat Statistics**[b]	
Number of elements	3,284,969
Length (bp) [% Genome]	731,540,381 [53.81%]
**Genome Annotation**[a]	
Protein coding genes	48,619
Functionally annotated	36,398 (74.86%)
Without functional annotation	12,221 (25.14%)
tRNA genes	33,934

[a]See also Supplementary S1-7.
[b]Summary of the RepeatMasker analysis (See also Supplementary Table S1-9).

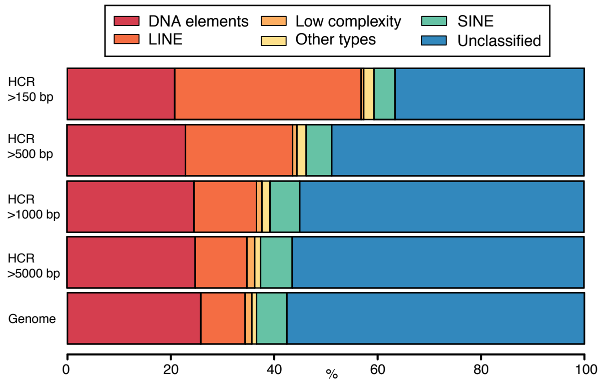

Figure 3. Bar plot of the annotation of the repetitive elements within the HCRs (2.5x threshold) at different intra-HCR length cutoffs (150, 500, 1,000 and 5,000 bp) (Supplementary Table S2-2a). Colors represent the type of repeat element identified by RepeatMasker. Other types class, include the LTR elements, Small RNA and Satellites information that represent a small fraction.

abundant interspersed repeats preventing genome continuity. Despite the low N50 we estimated that the draft presented here is mostly complete in terms of functional regions (see below).

Transcriptome assembly and genome annotation

We used the newly generated genome sequence to obtain a reference-guided assembly of the *D. silvatica* transcriptome with the RNAseq data from [39]. We used HISAT2 v2.1.0 (HISAT2, RRID:SCR_015530) [50] to map the RNAseq reads to the reference and Trinity v2.4.0. (Trinity, RRID:SCR_013048) [51] (genome guided bam, max intron = 50 kb, min coverage = 3) to assemble the transcriptome (named as Dsil-RefGuided transcriptome; Supplementary Table S1-10). We used the MAKER2 v2.31.9 (MAKER2, RRID:SCR_005309) [52] genome annotation pipeline for the structural annotation of *D. silvatica* genes (Supplementary Figure S2), using both *ab initio* gene predictions and annotation evidences from *D. silvatica* and other sources. For the *ab initio* gene predictions we initially trained Augustus v3.1.0 (Augustus, RRID:SCR_008417) [53] and SNAP (SNAP, RRID:SCR_002127) [54] softwares using scaffolds longer than 20 kb, and BUSCO gene models generated from completeness searches. Then we iteratively included a reliable set of proteins for a further training. This data set was composed by the 9,473 orthologs 1:1 identified in five *Dysdera* species and the 1:1 orthologs among spiders available at OrthoDB v10 (OrthoDB, RRID:SCR_011980) [55] (8,792). After several iterative training rounds, we applied MAKER2, Augustus and SNAP, adding other sources of evidence: i) transcript evidence (Dsil-RefGuided transcriptome), ii) RNAseq reads exon junctions generated with HISAT2 [50] and regtools [56] and, iii) proteins annotated in other arthropods, especially chelicerates (Figure 2; Supplementary Table S1-11). The annotation process resulted in 48,619 protein-coding and 33,934 tRNA genes. The mean Annotation Edit Distance (AED) upon protein-coding genes was 0.32 (Supplementary Figure S6), which is typical of a well-annotated genome [57, 58]. After each training and iterative annotation round, we checked the improvement of the annotation by means of the cumulative fraction of AED (Supplementary Table S1-12a; Supplementary Figure S7).

We searched for the presence of protein domain signatures in annotated protein-coding genes using InterProScan v5.15-54 (InterProScan, RRID:SCR_005829) [59, 60], which includes information from public databases (See additional details in Supplementary Table S1-7). Additionally, we used NCBI BLASTP v.2.4.0 (BLASTP, RRID:SCR_001010) [31] (E-value cutoff <10^{-5}; >75% alignment length) against the Swissprot database to annotate *D. silvatica* genes. We found that 74.9% (36,398 genes) of the predicted protein coding genes have hits with records of either InterPro (32,322 genes) (InterPro, RRID:SCR_006695) or Swissprot (17,225 cases) (Table 2; Supplementary Table S1-7).

Table 3. Completeness analysis[a].

BLAST Analysis[b]	# Identified (%)
Parasteatoda genes (*n* = 30,041)	19,580 (65.2%)
Single copy *Dysdera* (*n* = 9,473)	8,420 (88.9%)
Single copy Spiders (*n* = 2,198)	2,141 (97.4%)
CEG (*n* = 457)	438 (95.8%)
**BUSCO Analysis[c]**	
**Metazoa (*n* = 978)**	
Identified BUSCO	882 (90.2%)
Complete (C)	689 (70.5%)
Single copy (S)	662 (67.7%)
Duplicated (D)	27 (2.8%)
Fragmented (F)	193 (19.7%)
Missing (M)	96 (9.8%)
**Artrhopoda (*n* = 1,066)**	
Identified BUSCO	959 (89.9%)
Complete (C)	736 (69.1%)
Single copy (S)	702 (65.9%)
Duplicated (D)	34 (3.2%)
Fragmented (F)	223 (20.9%)
Missing (M)	107 (10.0%)

[a]Completeness analysis of the 36,398 functional annotated proteins of *D. silvatica*.
[b]BLASTP searches against different datasets. E-value cutoff < 10^{-3}, alignment length cutoff > 30% and identity cutoff > 30%.
[c]BUSCO analysis using default parameters against different datasets (BUSCO, RRID:SCR_015008)

Completeness

We determined the completeness of the *D. silvatica* genome assembly (Table 3) using BLASTP (E-value cutoff <10^{-3}; >30% of alignment length and identity > 50%). We searched for homologs of the functionally annotated peptides (36,398) in, i) among CEG genes of *Drosophila melanogaster* [41], ii) among the predicted peptides of *Parasteatoda tepidariorum*, a spider with a well annotated genome [61], iii) among the 9,473 1:1 orthologs across five *Dysdera* species and, iv) among the 2,198 single copy genes identified in all spiders and available in OrthoDB v10 [55]. We found in *D. silvatica* a high fraction of putative homologs (95.8% of CEG genes, and 97.4% spider-specific single copy genes (Table 3). Furthermore, the analysis based on the putative homologs of the single-copy genes included in the BUSCO data set (BUSCO, RRID:SCR_015008) [40], applying the default parameters for the genome and protein mode, also demonstrated the high completeness of the genome draft. Indeed the analysis recovered the ~90% of Metazoa or Arthropoda genes (v9), and nearly 70% of them are complete in *D. silvatica*.

We extended the search for *D. silvatica* homologs to a broader taxonomic range (Figure 2; Supplementary Table S1-11) by including other metazoan lineages and performing a series of local BLASTP searches (E-value cutoff < 10^{-3}; >30% align-

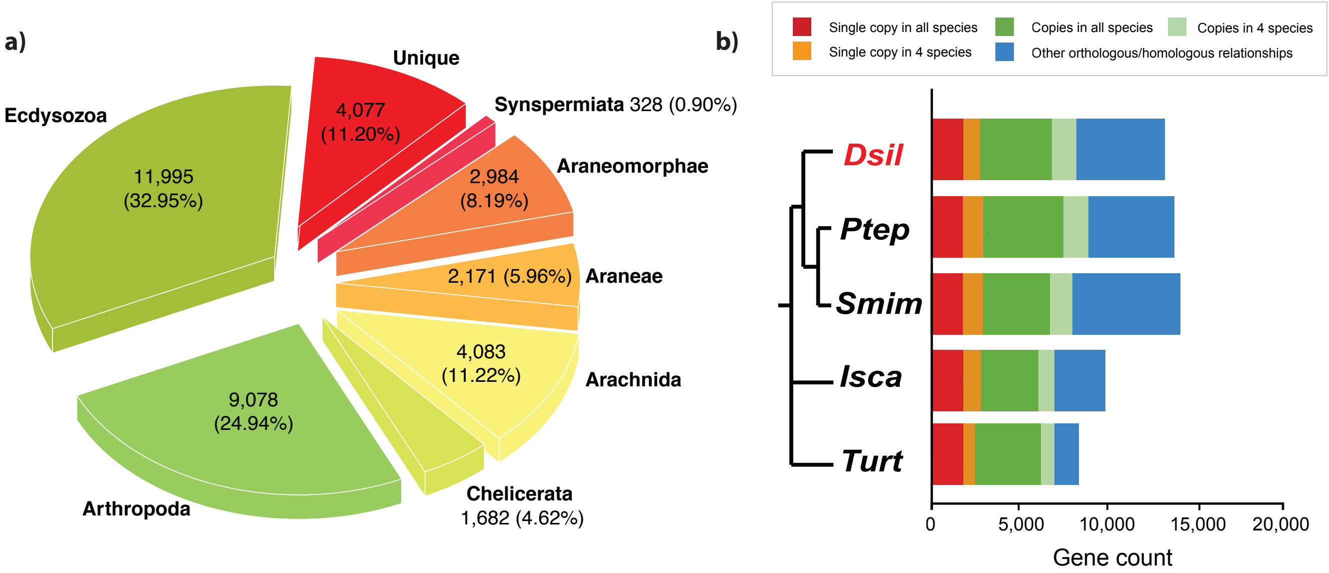

Figure 4. **a)** Pie chart illustrating the taxonomic distribution of positive BLAST hits of the *D. silvatica* protein-coding genes against the sequence data of species included in Figure 2. **b)** Homology relationships among *D. silvatica* (Dsil) and different chelicerates genomes available in OrthoDB v10 [55], *Parasteatoda tepidariorum* (Ptep), *Stegodyphus mimosarum* (Smim), *Ixodes scapularis* (Isca) and *Tetranychus urticae* (Turt). Red and orange bars indicate the fraction of single copy genes (1:1 orthologs) identified in all species, and in all but one (e.g., missing in one species), respectively. The dark and light green bar indicate the fraction of orthologs present in all species and in all but one, respectively, that are not included previous categories. The blue bar (other orthology/homology) shows other more complex homologous relationships. The results were generated uploading *D. silvatica* proteins to the OrthoDB web server.

ment length). We found that a great majority of *D. silvatica* genes are shared among arthropods (57.9%), being 11,995 of them (32.95%) also present in Ecdysozoa (Figure 4a). Remarkably, 9,560 genes appears to be spider-specific, being 4,077 of them specific (unique) of *D. silvatica*. Despite almost all these species-specific genes have interproscan signatures, the annotation metrics are poor compared with genes having homologs in other species (Supplementary Table S1-12b; Supplementary Figure S7 & S9); indeed, they have an average number of exons (2.8) and gene length (~168aa) which may reflect their partial nature. They could be part of a very large genes interspersed by repeats or complex sequences difficult to assemble. The analysis using OrthoDB (v10) [55] across five chelicerates (including *D. silvatica*) identified 1,798 genes, with an 1:1 orthologous relationships (Figure 4b), while 12,101 *D. silvatica* genes showed other more complex orthologous/homologous relationships (Figure 4b, Supplementary Table S1-12c & S3-1). The analysis across the genome annotations of some representative arthropods identified 950 genes with the 1:1 orthologous relationships (Supplementary Figure S8, Supplementary Table S1-12c & S3-2).

Mitochondrial genome assembly and annotation

We assembled the mitochondrial genome of *D. silvatica* (mtDsil) from 126,758 reads identified in the 100PE library by the software NOVOPlasty [62]. Our *de novo* assembly yielded a unique contig of 14,440 bp (coverage of 878X) (Supplementary Table S1-13). CGVIEW (CGVIEW, RRID:SCR_011779) [63] was used to generate a genome visualization of the annotated mtDsil genome (Supplementary Figure S10). We identified 2 rRNAs, 13 protein-coding genes and 15 tRNAs (out of the putative 22 tRNAs). Based on the contig length and the inability of standard automatic annotation algorithms to identify tRNA with missing arms, as reported for spiders [64], the complete set of tRNAs is most likely present for this species.

Conclusion

We have reported the assembly and annotation of the nuclear and mitochondrial genomes of the first representative of the spider superfamily Dysderoidea and the second genome of a Synspermiata, one of the main evolutionary lineages within the "true spiders" (Araneomorphae) and still sparsely sampled at the genomic level [14]. Despite the high coverage and the hybrid assembly strategy, the repetitive nature of the *D. silvatica* genome precluded obtaining a high continuity draft. The characteristic holocentric chromosomes of Dysderidae [17] may also explain the observed genome fragmentation; indeed, it has been recently shown that genome-wide centromere-specific repeat arrays are interspersed among euchromatin in holocentric plants (Rhynchospora, Cyperceae) [65].

Nevertheless, the completeness and the extensive annotations achieved for this genome, as well as the new reference-guided transcriptome, make this draft an excellent source tool for further functional and evolutionary analyses in this and other related species, including the origin and evolution of relevant spider traits, such as venom and silk. Moreover, the availability of new genomic information in a lineage with remarkable evolutionary features such as recurrent colonisations of the underground environment or complex reproductive anatomies indicatives of cryptic female choice, to cite two examples, will further provide valuable knowledge about relevant scientific questions, such as the molecular basis of adaptation to extreme habitats or the genetic drivers of sexual selection, along with more general aspects related to gene content across main arthropod groups, the consequences of whole genome duplications or phylogenetic relationships with the Araneae. Additionally, since this genus experienced a spectacular adaptive radiation in Canary Islands, current genome draft could be very useful to further studies understanding of the genomic basis of island radiations.

Availability of supporting data and materials

The whole genome shotgun project has been deposited at DDBJ/ENA/GenBank under accession number QLNU00000000 and project id PRJNA475203. The version described in this paper is version QLNU01000000. This project repository includes raw data, sequencing libraries information and assemblies of the mitochondrial and nuclear genomes. Other relevant datasets such as annotation, reference-guide assembled transcripts, repeat and HCR data, as other data relevant for the reproducibility of results are available in the GigaDB dataset [66].

Availability of supporting source code and requirements

The scripts employed and developed in this project are available under the github repository:

Project name: Genome assembly of *Dysdera silvatica*
Project home page: https://github.com/molevol-ub/Dysdera_silvatica_genome
Operating system(s): Platform independent
Programming language: Bash, Perl, Python, R
License: MIT

Abbreviations

AED: Annotation Edit Distance; BLAST: Basic Local Alignment Tool; bp: Base pair; BUSCO: Benchmarking Universal Single Copy Orthologs; CCS: Circularized Consensus sequence; CEG: Core Eukaryotic Genes; Cz: Cretaceous period; Dsil: *Dysdera silvatica*; Gb: Gigabase pairs; GC: Guanine Cytosine; GO: Gene Ontology; HCR: High Coverage Regions; Isca: *Ixodes scapularis*; kb: kilo base pairs; LINE: Long Interspersed Nuclear Element; LTR: Long Terminal Repeats; Mb: Mega base pairs; MP: Mate pair; Mya: Million years ago; NCBI: National Center for Biotechnology Information; PacBio: Pacific Bioscience; PE: Paired-End; Ptep: *Parasteatoda tepidariorum*; SINE: Short Interspersed Nuclear Element; Smim: *Stegodyphus mimosarum*; SMRT: Single-Molecule Real Time; Turt: *Tetranychus urticae*.

Competing interests

The authors declare that they have no competing interests.

Acknowledgements

We acknowledge the Garajonay National Parks for granting collection permits and helping with lodging and logistics during fieldwork. We also thank CNAG (Centro Nacional de Análisis Genómico) for the Nanopore sequencing facilities. This study was supported by the Ministerio de Economía y Competitividad of Spain (CGL2012-36863, CGL2013-45211 and CGL2016-75255), and by the Comissió Interdepartamental de Recerca I Innovació Tecnològica of Catalonia, Spain (2014SGR-1055 and 2014SGR1604). J.F.S.-H. was supported by a Formación del Profesor Universitario (FPU) grant (Ministerio de Educación of Spain, FPU13/0206). C.F.-L. by an IRBio PhD grant. S.H-A by Becas Postdoctorales en el Extranjero CONACyT. A.S.-G. by a Beatriu de Pinós grant (Generalitat de Catalunya, 2010-BP-B 00175), and J.R. and M.A.A. were partially supported by ICREA Academia (Generalitat de Catalunya).

Author's contribution

J.R., A.S.-G. and M.A.A designed the study. C.F.-L., J.F.S.-H., P.E. S.H-A. processed the samples and extracted DNA. J.F.S.-H. performed the bioinformatics analysis, and drafted the manuscript. J.F.S.-H., A.S.-G. and J.R. interpreted the data. All authors revised and approved the final manuscript.

References

1. World Spider Catalog, World Spider Catalog (2018).; 2018. http://wsc.nmbe.ch.
2. Pekár S, Toft S. Trophic specialisation in a predatory group: the case of prey-specialised spiders (Araneae). Biological Reviews 2015 aug;90(3):744–761. https://doi.org/10.1111/brv.12133.
3. Hopkin SP, Martin MH. Assimilation of Zinc, Cadmium, Lead, Copper, and Iron by the Spider Dysdera crocata, a Predator of Woodlice. Bull Environ Contam Toxicol 1985;34:183–187.
4. Pekár S, Líznarová E, Řezác M. Suitability of woodlice prey for generalist and specialist spider predators: a comparative study. Ecological Entomology 2016 apr;41(2):123–130. http://doi.wiley.com/10.1111/een.12285.
5. Toft S, Macías-Hernández N. Metabolic adaptations for isopod specialization in three species of <i>Dysdera</i> spiders from the Canary Islands. Physiological Entomology 2017 jun;42(2):191–198. http://doi.wiley.com/10.1111/phen.12192.
6. Řezáč M, Pekár S. Evidence for woodlice-specialization in Dysdera spiders: Behavioural versus developmental approaches. Physiological Entomology 2007;32(4):367–371. https://doi.org/10.1111/j.1365-3032.2007.00588.x.
7. Řezác M, Pekár S, Lubin Y. How oniscophagous spiders overcome woodlouse armour. Journal of Zoology 2008;275(1):64–71. https://doi.org/10.1111/j.1469-7998.2007.00408.x.
8. Arnedo MA, Oromí P, Ribera C. Radiation of the Spider Genus Dysdera (Araneae, Dysderidae) in the Canary Islands: Cladistic Assessment Based on Multiple Data Sets. Cladistics 2001;17:313–353. http://doi.wiley.com/10.1006/clad.2001.0168.
9. Macias-Hernandez N, de la Cruz López S, Roca-Cusachs M, Oromí P, Arnedo MA. A geographical distribution database of the genus Dysdera in the Canary Islands (Araneae, Dysderidae). ZooKeys 2016 oct;625(625):11–23. https://doi.org/10.3897/zookeys.625.9847.
10. Arnedo MA, Oromí P, Múrria C, Macías-Hernández N, Ribera C. The dark side of an island radiation: systematics and evolution of troglobitic spiders of the genus Dysdera Latreille (Araneae : Dysderidae) in the Canary Islands. Invertebrate Systematics 2007 jan;21(6):623. https://doi.org/10.1071/IS07015.
11. Michalik P, Ramírez MJ. Evolutionary morphology of the male reproductive system, spermatozoa and seminal fluid of spiders (Araneae, Arachnida) – Current knowledge and future directions. Arthropod Structure & Development 2014 jul;43(4):291–322. https://doi.org/10.1016/j.asd.2014.05.005.
12. Wheeler WC, Coddington JA, Crowley LM, Dimitrov D, Goloboff PA, Griswold CE, et al. The spider tree of life: phylogeny of Araneae based on target-gene analyses from an extensive taxon sampling. Cladistics 2017 dec;33(6):574–616. http://doi.wiley.com/10.1111/cla.12182.
13. Fernández R, Kallal RJ, Dimitrov D, Ballesteros JA, Arnedo MA, Giribet G, et al. Phylogenomics, Diversification Dynamics, and Comparative Transcriptomics across the Spi-

der Tree of Life. Current Biology 2018 may;28(9):1489–1497.e5. https://doi.org/10.1016/j.cub.2018.03.064.
14. Garb JE, Sharma PP, Ayoub NA. Recent progress and prospects for advancing arachnid genomics. Current Opinion in Insect Science 2018 feb;25:51–57. https://doi.org/10.1016/j.cois.2017.11.005.
15. Vizueta J, Rozas J, Sánchez-Gracia A. Comparative Genomics Reveals Thousands of Novel Chemosensory Genes and Massive Changes in Chemoreceptor Repertories across Chelicerates. Genome Biology and Evolution 2018 may;10(5):1221–1236. https://doi.org/10.1093/gbe/evy081.
16. Deeleman-Reinhold CL. The genus Rhode and the harpacteine genera Stalagtia, Folkia, Minotauria, and Kaemis (Araneae, Dysderidae) of Yugoslavia and Crete, with remarks on the genus Harpactea. Revue Arachnologique 1993;10(6):105–135.
17. Diaz MO, Maynard R, Brum-Zorrilla N. Diffuse Centromere and Chromosome Polymorphism in Haplogyne Spiders of the Families Dysderidae and Segestriidae. Cytogenetic and Genome Research 2010;128(1-3):131–138. https://doi.org/10.1159/000296273.
18. Uhl G. Two distinctly different sperm storage organs in female Dysdera erythrina (Araneae: Dysderidae). Arthropod structure & development 2000;29(2):163–169. https://doi.org/10.1016/S1467-8039(00)00019-0.
19. Burger M, Kropf C. Genital morphology of the haplogyne spider Harpactea lepida (Arachnida, Araneae, Dysderidae). Zoomorphology 2007 apr;126(1):45–52. https://doi.org/10.1007/s00435-007-0029-1.
20. Ballesteros JA, Sharma PP. A Critical Appraisal of the Placement of Xiphosura (Chelicerata) with Account of Known Sources of Phylogenetic Error. Systematic Biology 2019 mar;https://doi.org/10.1093/sysbio/syz011.
21. Lozano-Fernandez J, Tanner AR, Giacomelli M, Carton R, Vinther J, Edgecombe GD, et al. Increasing species sampling in chelicerate genomic-scale datasets provides support for monophyly of Acari and Arachnida. Nature Communications 2019;10:2295. https://doi.org/10.1038/s41467-019-10244-7.
22. Carlson DE, Hedin M. Comparative transcriptomics of Entelegyne spiders (Araneae, Entelegynae), with emphasis on molecular evolution of orphan genes. PLOS ONE 2017 apr;12(4):e0174102. https://doi.org/10.1371/journal.pone.0174102.
23. Gregory TR, Animal Genome Size Database; 2018. http://www.genomesize.com.
24. Hare EE, Johnston JS. Genome Size Determination Using Flow Cytometry of Propidium Iodide-Stained Nuclei. Humana Press; 2012.p. 3–12. https://doi.org/10.1007/978-1-61779-228-1_1.
25. Rasch EM, Connelly BA. Genome size and endonuclear DNA replication in spiders. Journal of Morphology 2005 aug;265(2):209–214. http://doi.wiley.com/10.1002/jmor.10352.
26. Gregory TR, Shorthouse DP. Genome Sizes of Spiders. Journal of Heredity 2003 jul;94(4):285–290. https://doi.org/10.1093/jhered/esg070.
27. Dpooležel J, Binarová P, Lcretti S. Analysis of Nuclear DNA content in plant cells by Flow cytometry. Biologia Plantarum 1989 mar;31(2):113–120. https://doi.org/10.1007/BF02907241.
28. Marçais G, Kingsford C. A fast, lock-free approach for efficient parallel counting of occurrences of k-mers. Bioinformatics 2011 mar;27(6):764–770. https://doi.org/10.1093/bioinformatics/btr011.
29. Vurture GW, Sedlazeck FJ, Nattestad M, Underwood CJ, Fang H, Gurtowski J, et al. GenomeScope: fast reference-free genome profiling from short reads. Bioinformatics 2017 jul;33(14):2202–2204. https://doi.org/10.1093/bioinformatics/btx153.
30. Austin CM, Tan MH, Harrisson KA, Lee YP, Croft LJ, Sunnucks P, et al. De novo genome assembly and annotation of Australia's largest freshwater fish, the Murray cod (Maccullochella peelii), from Illumina and Nanopore sequencing read. GigaScience 2017 aug;6(8):1–6. https://doi.org/10.1093/gigascience/gix063.
31. Altschul SF, Gish W, Miller W, Myers EW, Lipman DJ. Basic local alignment search tool. Journal of molecular biology 1990 oct;215(3):403–10. https://doi.org/10.1016/S0022-2836(05)80360-2.
32. Schmieder R, Edwards R. Fast identification and removal of sequence contamination from genomic and metagenomic datasets. PloS one 2011 jan;6(3):e17288. https://doi.org/10.1093/bioinformatics/btr026.
33. Bolger AM, Lohse M, Usadel B. Trimmomatic: a flexible trimmer for Illumina sequence data. Bioinformatics 2014 aug;30(15):2114–2120. https://doi.org/10.1093/bioinformatics/btu170.
34. O'Connell J, Schulz-Trieglaff O, Carlson E, Hims MM, Gormley NA, Cox AJ. NxTrim: Optimized trimming of Illumina mate pair reads. Bioinformatics 2015;31(12):2035–2037. https://doi.org/10.1093/bioinformatics/btv057.
35. PacBio, Single Molecule Real Time (SMRT);. https://www.pacb.com/products-and-services/analytical-software/smrt-analysis/.
36. Walker BJ, Abeel T, Shea T, Priest M, Abouelliel A, Sakthikumar S, et al. Pilon: An Integrated Tool for Comprehensive Microbial Variant Detection and Genome Assembly Improvement. PLoS ONE 2014 nov;9(11):e112963. https://doi.org/10.1371/journal.pone.0112963.
37. Zimin AV, Marçais G, Puiu D, Roberts M, Salzberg SL, Yorke JA. The MaSuRCA genome assembler. Bioinformatics (Oxford, England) 2013 nov;29(21):2669–77. https://doi.org/10.1093/bioinformatics/btt476.
38. Zhang SV, Zhuo L, Hahn MW. AGOUTI: improving genome assembly and annotation using transcriptome data. GigaScience 2016 dec;5(1):31. https://doi.org/10.1186/s13742-016-0136-3.
39. Vizueta J, Frias-López C, Macías-Hernández N, Arnedo MA, Sánchez-Gracia A, Rozas J, et al. Evolution of chemosensory gene families in arthropods: Insight from the first inclusive comparative transcriptome analysis across spider appendages. Genome Biology and Evolution 2017 dec;9(1):178–196. https://doi.org/10.1093/gbe/evw296.
40. Simão FA, Waterhouse RM, Ioannidis P, Kriventseva EV, Zdobnov EM. BUSCO: Assessing genome assembly and annotation completeness with single-copy orthologs. Bioinformatics 2015;31(19):3210–3212. https://doi.org/10.1093/bioinformatics/btv351.
41. Parra G, Bradnam K, Ning Z, Keane T, Korf I. Assessing the gene space in draft genomes. Nucleic Acids Research 2009 jan;37(1):289–297. https://doi.org/10.1093/nar/gkn916.
42. Li H, Handsaker B, Wysoker A, Fennell T, Ruan J, Homer N, et al. The Sequence Alignment/Map format and SAMtools. Bioinformatics (Oxford, England) 2009 aug;25(16):2078–9. https://doi.org/10.1093/bioinformatics/btp352.
43. Langmead B, Salzberg SL. Fast gapped-read alignment with Bowtie 2. Nature methods 2012 apr;9(4):357–9. http://dx.doi.org/10.1038/nmeth.1923.
44. Li H. Minimap2: pairwise alignment for nucleotide sequences. Bioinformatics 2018 sep;34(18):3094–3100. https://doi.org/10.1093/bioinformatics/bty191.
45. Smit, AF; Hubley R, RepeatModeler Open-1.0. 2008-2015;. http://www.repeatmasker.org.

46. Smit, AF; Hubley, R ; Green P, RepeatMasker Open-3.0. 1996-2010;. http://www.repeatmasker.org.
47. Wheeler TJ, Clements J, Eddy SR, Hubley R, Jones TA, Jurka J, et al. Dfam: a database of repetitive DNA based on profile hidden Markov models. Nucleic Acids Research 2012 nov;41(D1):D70–D82. https://doi.org/10.1093/nar/gks1265.
48. Bao W, Kojima KK, Kohany O. Repbase Update, a database of repetitive elements in eukaryotic genomes. Mobile DNA 2015 dec;6(1):11. https://doi.org/10.1186/s13100-015-0041-9.
49. Jurka J, Kapitonov VV, Pavlicek A, Klonowski P, Kohany O, Walichiewicz J. Repbase Update, a database of eukaryotic repetitive elements. Cytogenetic and genome research 2005;110(1-4):462–7. https://doi.org/10.1159/000084979.
50. Kim D, Langmead B, Salzberg SL. HISAT: a fast spliced aligner with low memory requirements. Nature Methods 2015 apr;12(4):357–360. https://doi.org/10.1038/nmeth.3317.
51. Haas BJ, Papanicolaou A, Yassour M, Grabherr M, Blood PD, Bowden J, et al. De novo transcript sequence reconstruction from RNA-seq using the Trinity platform for reference generation and analysis. Nature protocols 2013 aug;8(8):1494–512. http://dx.doi.org/10.1038/nprot.2013.084.
52. Holt C, Yandell M. MAKER2: an annotation pipeline and genome-database management tool for second-generation genome projects. BMC Bioinformatics 2011 dec;12(1):491. https://doi.org/10.1186/1471-2105-12-491.
53. Stanke M, Steinkamp R, Waack S, Morgenstern B. AUGUSTUS: a web server for gene finding in eukaryotes. Nucleic acids research 2004 jul;32(Web Server issue):W309–12. https://doi.org/10.1093/nar/gki458.
54. Korf I. Gene finding in novel genomes. BMC Bioinformatics 2004 may;5(1):59. https://doi.org/10.1186/1471-2105-5-59.
55. Kriventseva EV, Kuznetsov D, Tegenfeldt F, Manni M, Dias R, Simão FA, et al. OrthoDB v10: sampling the diversity of animal, plant, fungal, protist, bacterial and viral genomes for evolutionary and functional annotations of orthologs. Nucleic Acids Research 2019 jan;47(D1):D807–D811. https://doi.org/10.1093/nar/gky1053.
56. Feng YY, Ramu A, Cotto KC, Skidmore ZL, Kunisaki J, Conrad DF, et al. RegTools: Integrated analysis of genomic and transcriptomic data for discovery of splicing variants in cancer. bioRxiv 2018 nov;p. 436634. https://www.biorxiv.org/content/10.1101/436634v2.
57. Eilbeck K, Moore B, Holt C, Yandell M. Quantitative measures for the management and comparison of annotated genomes. BMC Bioinformatics 2009 feb;10(1):67. https://doi.org/10.1186/1471-2105-10-67.
58. Yandell M, Ence D. A beginner's guide to eukaryotic genome annotation. Nature reviews Genetics 2012 may;13(5):329–42. https://doi.org/10.1038/nrg3174.
59. InterPro in 2019: improving coverage, classification and access to protein sequence annotations. Nucleic Acids Research 2019 jan;47(D1):D351–D360. https://doi.org/10.1093/nar/gky1100.
60. InterProScan 5: genome-scale protein function classification. Bioinformatics 2014 may;30(9):1236–1240. https://doi.org/10.1093/bioinformatics/btu031.
61. Schwager EE, Sharma PP, Clarke T, Leite DJ, Wierschin T, Pechmann M, et al. The house spider genome reveals an ancient whole-genome duplication during arachnid evolution. BMC Biology 2017 dec;15(1):62. https://doi.org/10.1186/s12915-017-0399-x.
62. Dierckxsens N, Mardulyn P, Smits G. NOVOPlasty: de novo assembly of organelle genomes from whole genome data. Nucleic Acids Research 2016 oct;45(4):gkw955. https://doi.org/10.1093/nar/gkw955.
63. Stothard P, Wishart DS. Circular genome visualization and exploration using CGView. Bioinformatics 2005 feb;21(4):537–539. https://doi.org/10.1093/bioinformatics/bti054.
64. Masta SE, Boore JL. The Complete Mitochondrial Genome Sequence of the Spider Habronattus oregonensis Reveals Rearranged and Extremely Truncated tRNAs. Molecular Biology and Evolution 2004 jan;21(5):893–902. https://doi.org/10.1093/molbev/msh096.
65. Marques A, Ribeiro T, Neumann P, Macas J, Novák P, Schubert V, et al. Holocentromeres in Rhynchospora are associated with genome-wide centromere-specific repeat arrays interspersed among euchromatin. Proceedings of the National Academy of Sciences of the United States of America 2015 nov;112(44):13633–8. https://doi.org/10.1073/pnas.1512255112.
66. Sánchez-Herrero JF, Frías-López C, Escuer P, Hinojosa-Alvarez S, Arnedo MA, Sánchez-Gracia A, et al. Supporting data for "The draft genome sequence of the spider Dysdera silvatica (Araneae, Dysderidae): A valuable resource for functional and evolutionary genomic studies in chelicerates". GigaScience Database 2019;http://dx.doi.org/10.5524/100628.

 Click here to access/download;Table;Tables.xlsx

Table 1: Sequencing data and library information.

Run ID	Library	Insert Size	Read Lengths	Lanes	Total Bases	Raw Read Pairs	Coverage[a]
PE	Illumina HiSeq200 - Truseq	370 bp	100x100 PE	1	51,202,445,102	506,954,902	30X
MP	Illumina HiSeq200 - Nextera	5 kb	100x100 PE	1	39,609,522,995	392,173,495	23X
Nanopore	Nanopore 1D Libraries	-	Nanopore	5	23,193,357,481	20,534,058	14X
PacBio	PacBio RSII 20kb SMRTbell	-	SMRT	8	9,652,844,880	1,455,288	6X

[a]Based on the genome size estimated by flow cytometry (1.7 Gb)

Table 2: *Dysdera silvatica* nuclear genome assembly and annotation statistics

**Genome Assembly[a]**	
**Assembly Size (bp)**	1,359,336,805
**% AT / CG / N**	64.91% / 34.83% / 0.26%
**Number of Scaffolds**	65,205
**Longest scaffold**	340,047
**N50**	38,017
**L50**	10,436
**Repeat Statistics[b]**	
**Number of elements**	3,284,969
**Length (bp) [% Genome]**	731,540,381 [53.81%]
**Genome Annotation[a]**	
**Protein coding genes**	48,619
**Functionally annotated**	36,398 (74.86%)
**Without functional annotation**	12,221 (25.14%)
**tRNA genes**	33,934

[a] See also Supplementary S1-7

[b] Summary of the RepeatMasker analysis (See also Supplementary Table S1-9).

Table 3: Completeness Analysis

BLAST Analysis[b]		# Identified (%)
	**Parasteatoda genes (*n* = 30,041)**	19,580 (65.2%)
	**Single copy Dysdera (*n* = 9,473)**	8,420 (88.9%)
	**Single copy Spiders (*n* = 2,198)**	2,141 (97.4%)
	**CEG (*n* = 457)**	438 (95.8%)

BUSCO Analysis[c]		
**Metazoa (*n* = 978)**	**Identified BUSCO**	882 (90.2%)
	**Complete (C)**	689 (70.5%)
	**Single copy (S)**	662 (67.7%)
	**Duplicated (D)**	27 (2.8%)
	**Fragmented (F)**	193 (19.7%)
	**Missing (M)**	96 (9.8%)
**Artrhopoda (*n* =1,066)**	**Identified BUSCO**	959 (89.9%)
	**Complete (C)**	736 (69.1%)
	**Single copy (S)**	702 (65.9%)
	**Duplicated (D)**	34 (3.2%)
	**Fragmented (F)**	223 (20.9%)
	**Missing (M)**	107 (10.0%)

[a]Completenes analysis of the 36,398 functional annotated proteins of *D. silvatica*
[b]BLASTP searches against different datasets. E-value cutoff < 10^{-3}, alignment length cutoff > 30% and identity cutoff > 30%.
[c]BUSCO analysis using default parameters against different datasets

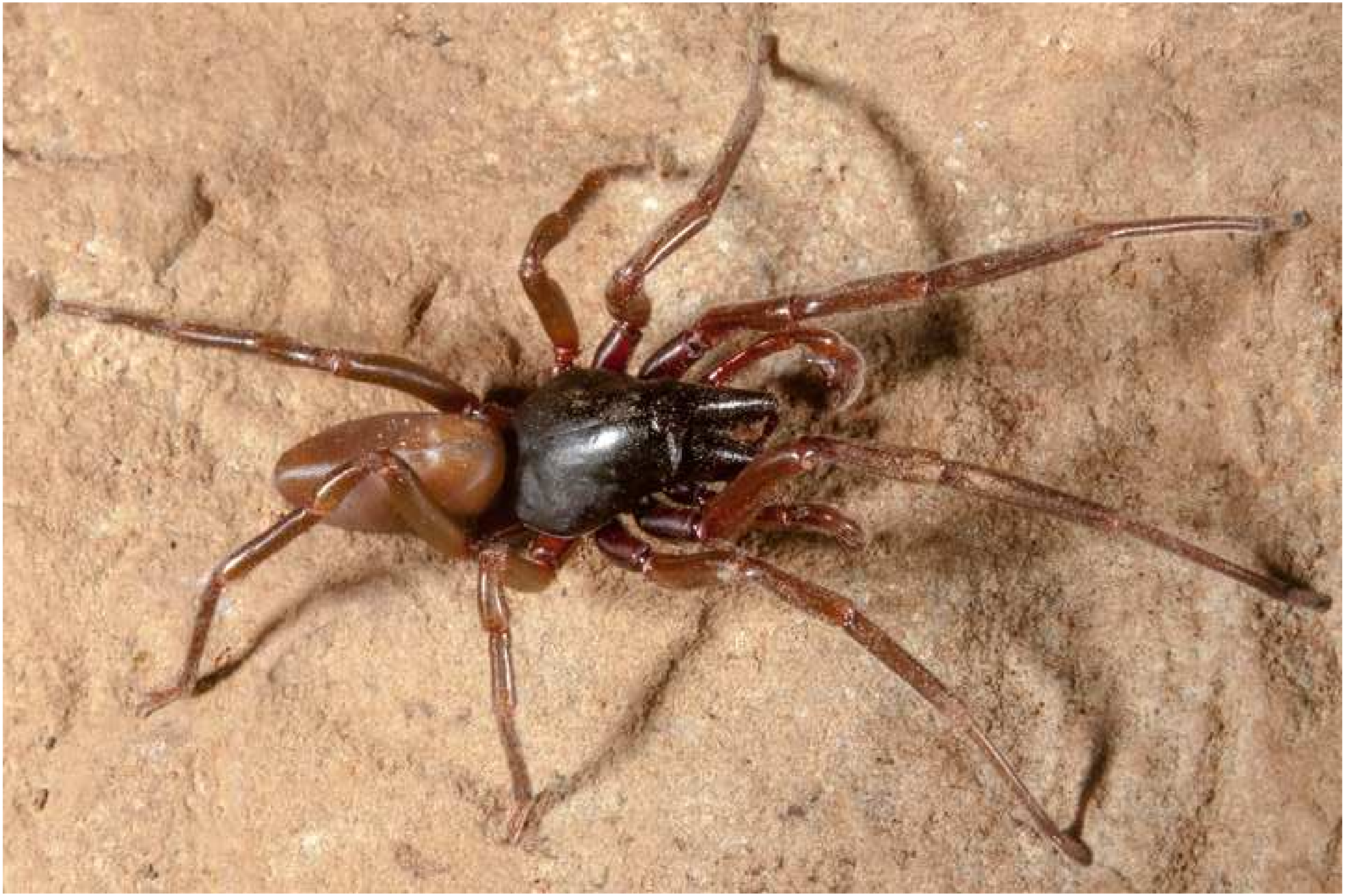

Figure2

Click here to access/download;Figure;Figure2.pdf

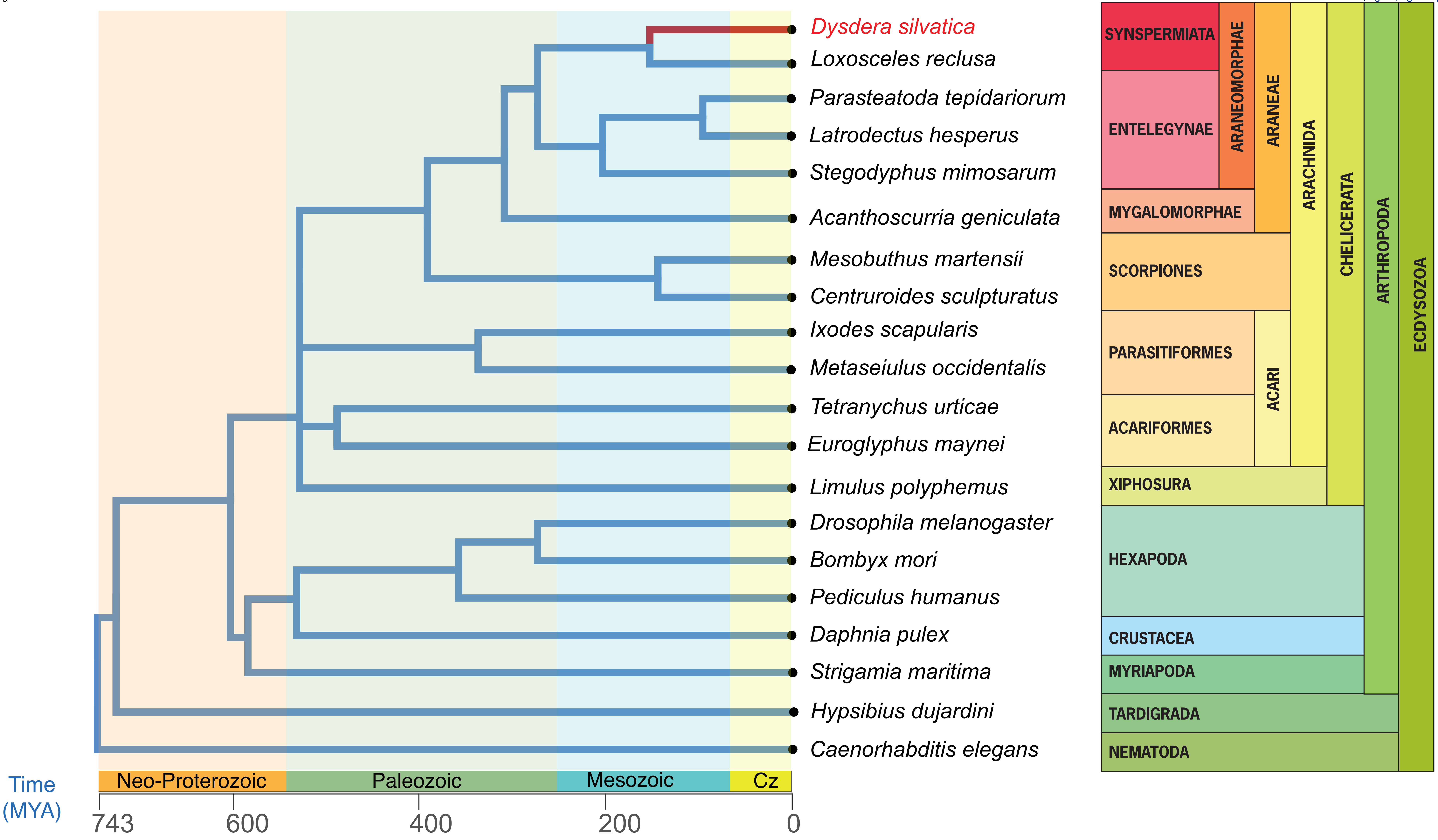

Figure3

Click here to access/download;Figure;Figure3.pdf

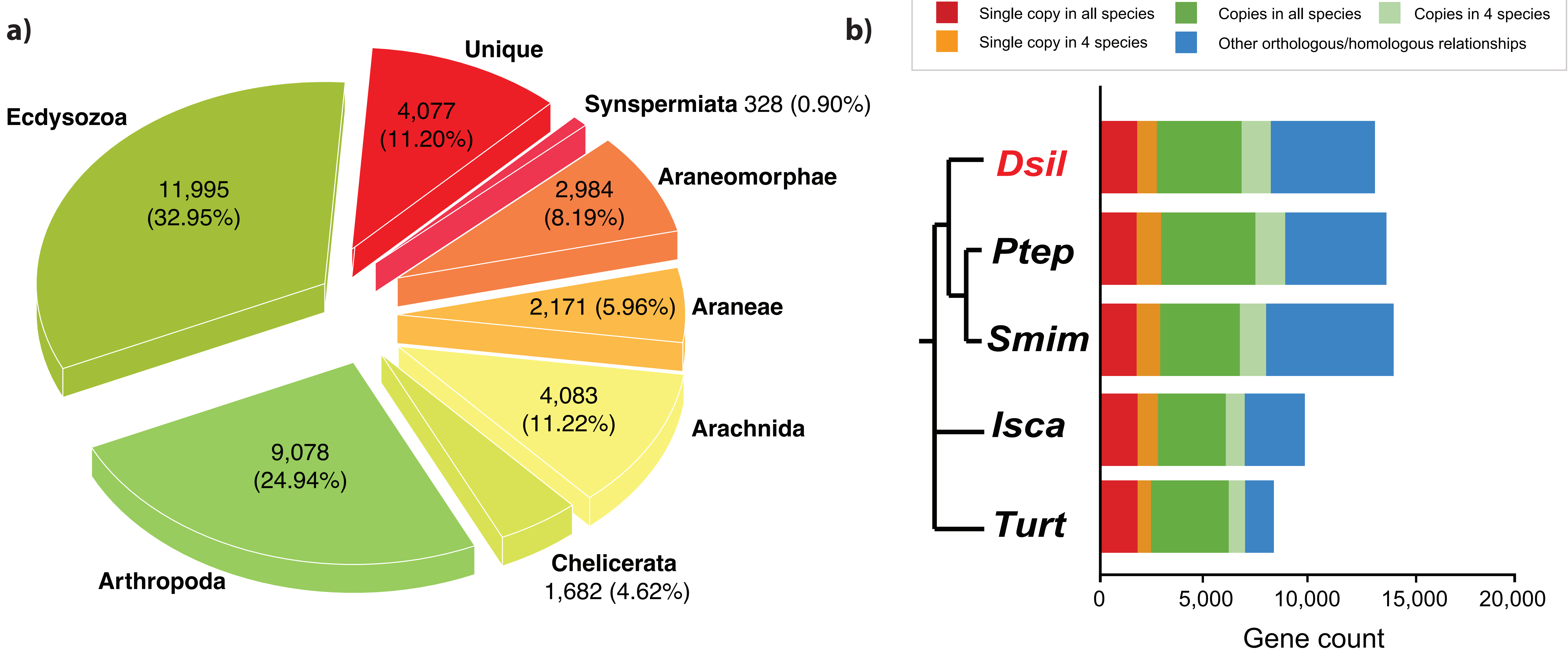

Figure4
Click here to access/download;Figure;Figure4.pdf

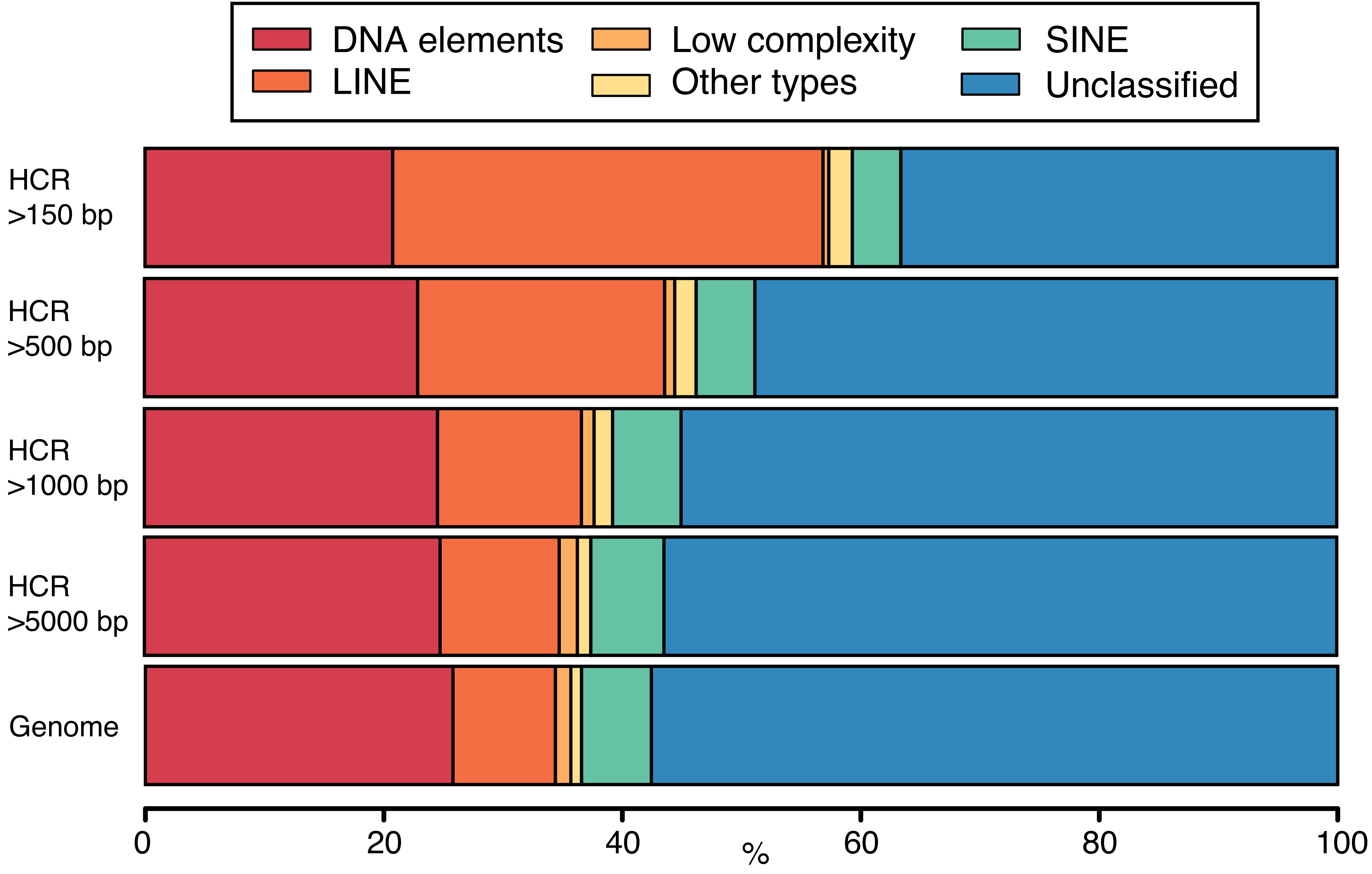

Click here to access/download
Supplementary Material
SanchezHerrero_Dsilvatica_SupMaterial_Summary.pdf

Sup Table1

Click here to access/download
Supplementary Material
20190625_Supplementary_Table_1.xlsx

Sup Table2

Click here to access/download
Supplementary Material
20190506_Supplementary_Table_2.xlsx

Sup Table3

Click here to access/download
Supplementary Material
20190625_Supplementary_Table_3.xlsx

Figure S1

Click here to access/download
Supplementary Material
Figure_S1.png

Figure S2

Click here to access/download
Supplementary Material
Figure_S2.pdf

Figure S3

Click here to access/download
Supplementary Material
Figure_S3.pdf

Figure S4

Click here to access/download
Supplementary Material
Figure_S4.pdf

Figure S5a

Click here to access/download
Supplementary Material
Figure_S5a.pdf

Figure S5b

Click here to access/download
Supplementary Material
Figure_S5b.pdf

Figure S6

Click here to access/download
Supplementary Material
Figure_S6.pdf

Figure S7

Click here to access/download
Supplementary Material
Figure_S7.pdf

Figure S8

Click here to access/download
Supplementary Material
Figure_S8.pdf

Figure S9

Click here to access/download
Supplementary Material
Figure_S9.pdf

Figure S10

Click here to access/download
Supplementary Material
Figure_S10.pdf

Cover Click here to access/download;Personal Cover;CoverLetter_GIGA.pdf

UNIVERSITAT DE BARCELONA

Dr. Julio Rozas
Catedràtic de Genètica

Departament de Genètica, Microbiologia i Estadística
Facultat de Biologia

Diagonal 643
Edifici Prevosti
08028 Barcelona
Spain

Tel. +34 93 4021495
Fax. +34 93 4034420
jrozas@ub.edu
www.ub.edu/molevol/julio

July 17, 2019

GigaScience

Dear Sirs,

Please find enclosed the revised version of our manuscript GIGA-D-19-00156R2 entitled "The draft genome sequence of the spider *Dysdera silvatica* (Araneae, Dysderidae): A valuable resource for functional and evolutionary genomic studies in chelicerates" in which we have fixed some points in your annotated version.

In particular

For the numbers in the text and tables, you wrote like this "39 609 522 995", please replace the space with comma in all numbers.
Done

And you can also revise the following things this time:
1) RRIDs for software: eg. you wrote "with Jellyish v.2.2.3 (RRID:SCR_005491) [28].", it should be "with Jellyish v.2.2.3 (Jellyish, RRID:SCR_005491) [28]." Please revise all these writings.
Done

2) For the QIAGEN protocol issue Scott mentioned in last email, if you think it's a better way to present the protocol, you can add it this time.
I think there is no need. We have used the original protocol of QIAGEN, with some very minor adjustments that are also indicated in the paper (extra wash; one centrifugation step less). Moreover, there is no any entry in protocols.io for the original QUIAGEN.

With best regards,

Sincerely,

Dr. Julio Rozas
Professor of Genetics
