# Supplementary material for: The draft genome sequence of the spider Dysdera silvatica (Araneae, Dysderidae): A valuable resource for functional and evolutionary genomic studies in chelicerates
Source: Gigascience. 2019 Aug 20;8(8):giz099. doi: 10.1093/gigascience/giz099 (PMC6701490; doi:10.1093/gigascience/giz099)
Supplement: giz099_Supplemental_Files [file giz099_supplemental_files.zip › SanchezHerrero_Dsilvatica_SupMaterial_Summary.pdf]

# The draft genome sequence of the spider *Dysdera silvatica* (Araneae, Dysderidae): A valuable resource for functional and evolutionary genomic studies in chelicerates

José Francisco Sánchez-Herrero[1,2], Cristina Frías-López[1,2], Paula Escuer[1,2], Silvia Hinojosa-Alvarez[1,2,3], Miquel A. Arnedo[1,4], Alejandro Sánchez-Gracia[1,2,*] and Julio Rozas[1,2,*]

[1]Departament de Genètica, Microbiologia i Estadística, Universitat de Barcelona (UB), Barcelona, Spain
[2]Institut de Recerca de la Biodiversitat (IRBio) (UB)
[3]Jardín Botánico, Instituto de Biología, Universidad Nacional Autónoma de México, Ciudad de México, México
[4]Departament de Biologia Evolutiva, Ecologia i Ciències Ambientals (UB)

## Additional Files

### SUPLEMENTARY FIGURES

**Supplementary Figure 1:** GenomeScope *k-mer* profile plot for the *D. silvatica* genome *Dsil v1.2*, based on 21-*mers* of the PE reads. The observed *k-mer* frequency distribution is depicted in blue, whereas the GenomeScope fit model is shown as a black line. The unique and putative error k-*mer* distributions are plotted in yellow and red, respectively.

**Supplementary Figure 2:** Schematic representation of the hierarchical workflow used to generate the assembly of the *D. silvatica* genome.

**Supplementary Figure 3:** Genome coverage distribution for the different genome sequencing data used in this study. Dash lines indicate the mean genome coverage for the particular sequencing technology.

**Supplementary Figure 4:** Analysis of the number and distribution of the High Coverage Regions (HCR) across the genome. **a)** Schematic representation of the genome coverage distribution along a contig (~35 kb). The pink dotted line denotes the mean genome coverage estimated for PE read library (Supplementary Table S1-9) (~30X). The green and orange dotted lines reflect, 2.5x and 5x thresholds, respectively, of the average coverage (75X and 150X, respectively). The intra HCR length (in blue) reflects the physical distance fulfilling the threshold coverage (2.5x or 5x), while the Inter HCR (red) denotes the distance between HCRs. **b)** Frequency distribution of the intra-HCR length (blue) and inter-HCR (red) across the 34 937 contigs for the 2.5x (green) or 5x (orange) times the average coverage (See Supplementary Table 2-2 and Supplementary File for details). The minimum value for any inter-HCR was always >10bp. The yellow line denotes the mean distribution value.

**Supplementary Figure 5:** Frequency distribution of the intra-HCR length (blue) and inter-HCR (red) for different length cutoffs (150, 500, 1 000 and 5 000) across the 34 937 contigs for the 2.5x (a) or 5x (b) threshold coverage (See Supplementary Table 2-2 and Supplementary File for details). The minimum value for any inter-HCR was >10bp. The yellow line denotes the mean distribution value.

**Supplementary Figure 6:** Bar plot of the annotation of the repetitive elements within the HCRs (5x threshold) at different intra-HCR length cutoffs (150, 500, 1 000 and 5 000 bp) (Supplementary Table S2-2a). Colors represent the type of repeat element identified by RepeatMasker. Other types class, include the LTR elements, Small RNA and Satellites information that represent a small fraction.

**Supplementary Figure 7:** Cumulative fraction of the frequency distribution of the Annotation Edit Distance (AED) provided by MAKER2 for different steps of the annotation process (Supplementary Table S1-12). The two iterative training rounds (R1 and R2) are shown in dashed blue. The final test (F) rounds are depicted by green lines: F1 using only *D. silvatica* transcripts and F2 using proteins from a broad taxonomic range (Figure 2; Supplementary Table S11). The F2 line shows the cumulative fraction of annotation for the final 48,619 protein-coding genes annotated with an average AED of 0.32. The red and orange dashed lines, represent the cumulative fraction of annotation for the 36,398 functionally annotated protein-coding genes (AED value of 0.268), and for the 4 077 unique *Dysdera silvatica* genes (AED of 0.4), respectively. The AED value is a direct measure of the annotation quality and its values range from 0 (high evidence and exact match based on alignment) to 1 (no evidence support).

**Supplementary Figure 8:** Homologous relationships between *D. silvatica* (Dsil) and five representative metazoan genomes available in OrthoDB v10 database (Kriventseva 2019): *Strigamia maritima* (Smar), *Drosophila melanogaster* (Dmel), *Limulus polyphemus* (Lpol), *Ixodes scapularis* (Isca), *Parasteatoda tepidariorum* (Ptep) and *D. silvatica* (Dsil). Red and orange bars indicate the fraction of single copy genes (1:1 orthologs) identified in all species, and in all but one (eg, missing in one species), respectively. The dark and light green bar indicates the fraction of orthologs present in all species and in all but one, respectively, that are not included previous categories. The blue bar (other orthology/homology) shows other more complex homologous relationships. The results were generated uploading *D. silvativa* proteins to the OrthoDB web server.

**Supplementary Figure S9:** Cumulative fraction of frequency distribution of covered exon overlap match by RNAseq or ab initio evidence at the splice site (dash lines) or exon level (solid lines) for the different datasets (in colours: red for species-specific proteins; green for functionally annotated proteins and blue for all structurally annotated proteins) (Supplementary Table S1-12b).

**Supplementary Figure S10:** Structure and functional annotation of the mitochondrial genome.

## SUPLEMENTARY TABLES

**Supplementary Table S1-1:** Collection of samples used in these study.

**Supplementary Table S1-2:** DNA sequencing read files used in this study.

**Supplementary Table S1-3:** NCBI data used for the contaminant search step.

**Supplementary Table S1-4:** Pre-processing statistics for each library.

**Supplementary Table S1-5:** Samples used for the RNAseq study.

**Supplementary Table S1-6:** RNA sequencing read files.

**Supplementary Table S1-7:** Genome descriptive statistics

**Supplementary Table S1-8:** Coverage analysis.

**Supplementary Table S1-9:** RepeatMasker analysis of *D. silvatica* genome.

**Supplementary Table S1-10:** Reference-guided transcriptome assembly statistics.

**Supplementary Table S1-11:** Source of proteins to conduct the annotation of *D. silvatica* genes and completeness analysis.

**Supplementary Table S1-12:** Annotation statistics.

**Supplementary Table S1-13:** Mitochondrial assembly metrics and annotation features

**Supplementary Table S2-1:** Example of results for the High Coverage Region (HCR) analysis.

**Supplementary Table S2-2:** High Coverage Region (HCR) descriptive statistics.

**Supplementary Table S2-3:** Enrichment analysis of the intersection of High Coverage regions (HCRs) with RepeatMasker annotation (main repeats).

**Supplementary Table S2-4:** Enrichment analysis of the intersection of High Coverage regions (HCRs) (2.5x mean coverage threshold) with RepeatMasker annotation (subtypes of main repeats).

**Supplementary Table S2-5:** Enrichment analysis of the intersection of High Coverage regions (HCRs) (5x mean coverage threshold) with RepeatMasker annotation (subtypes of main repeats).

**Supplementary Table S3-1:** List of the *D. silvatica* genes identified in the OrthoDB analysis across five chelicerates.

**Supplementary Table S3-2:** List of the *D. silvatica* genes identified in the OrthoDB analysis across six arthropods.

**SUPLEMENTARY FILES**

**Supplementary File SF1**: Additional data and results including the mapping coverage distribution results, High Coverage Region (HCR) analysis, Annotation Edit distance (AED) statistics and OrthoDB comparative results.
