# Supplementary figures and images for: The draft genome sequence of the spider Dysdera silvatica (Araneae, Dysderidae): A valuable resource for functional and evolutionary genomic studies in chelicerates
Source: Gigascience. 2019 Aug 20;8(8):giz099. doi: 10.1093/gigascience/giz099 (PMC6701490; doi:10.1093/gigascience/giz099)

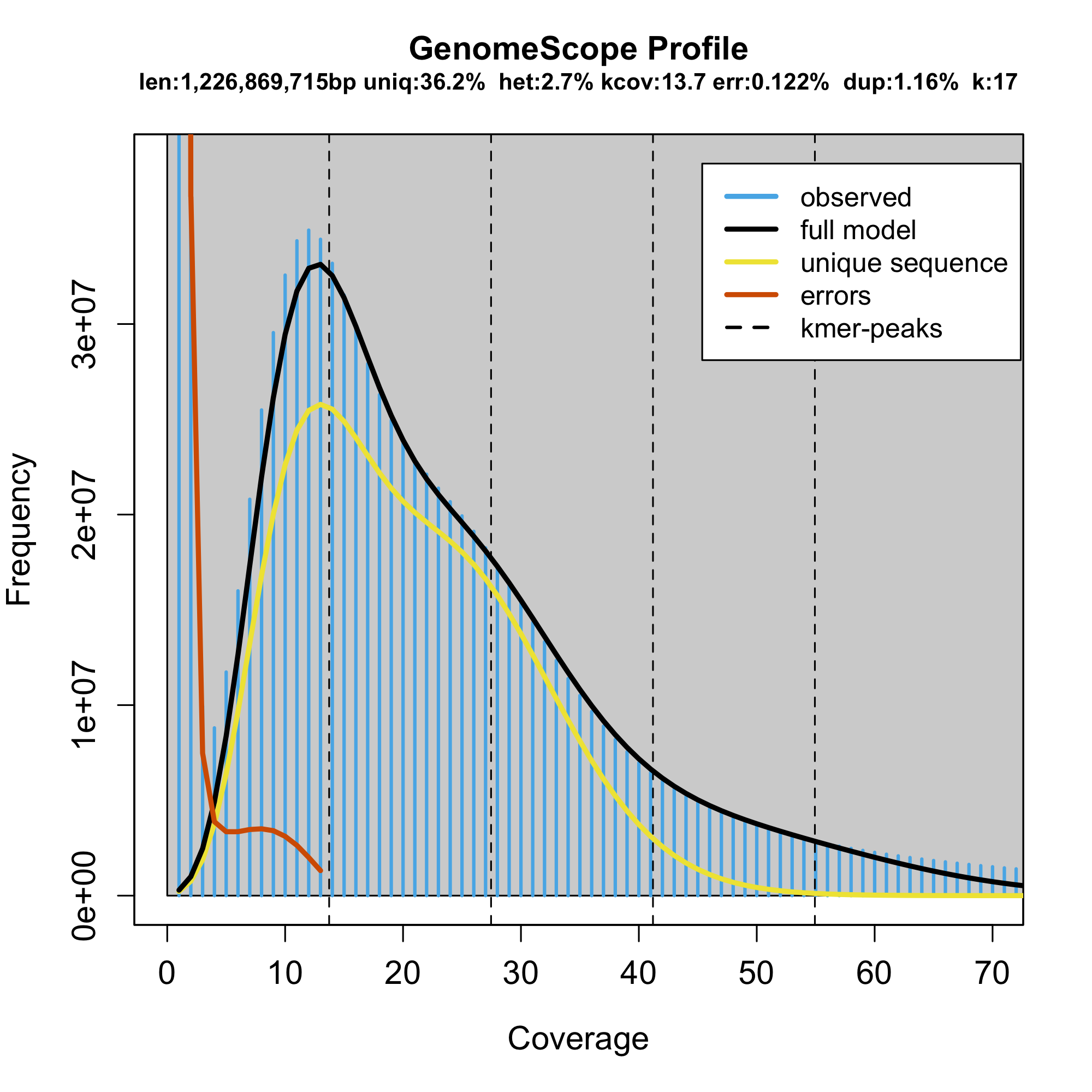

Supplement: giz099_Supplemental_Files [file giz099_supplemental_files.zip › Figure_S1.png]

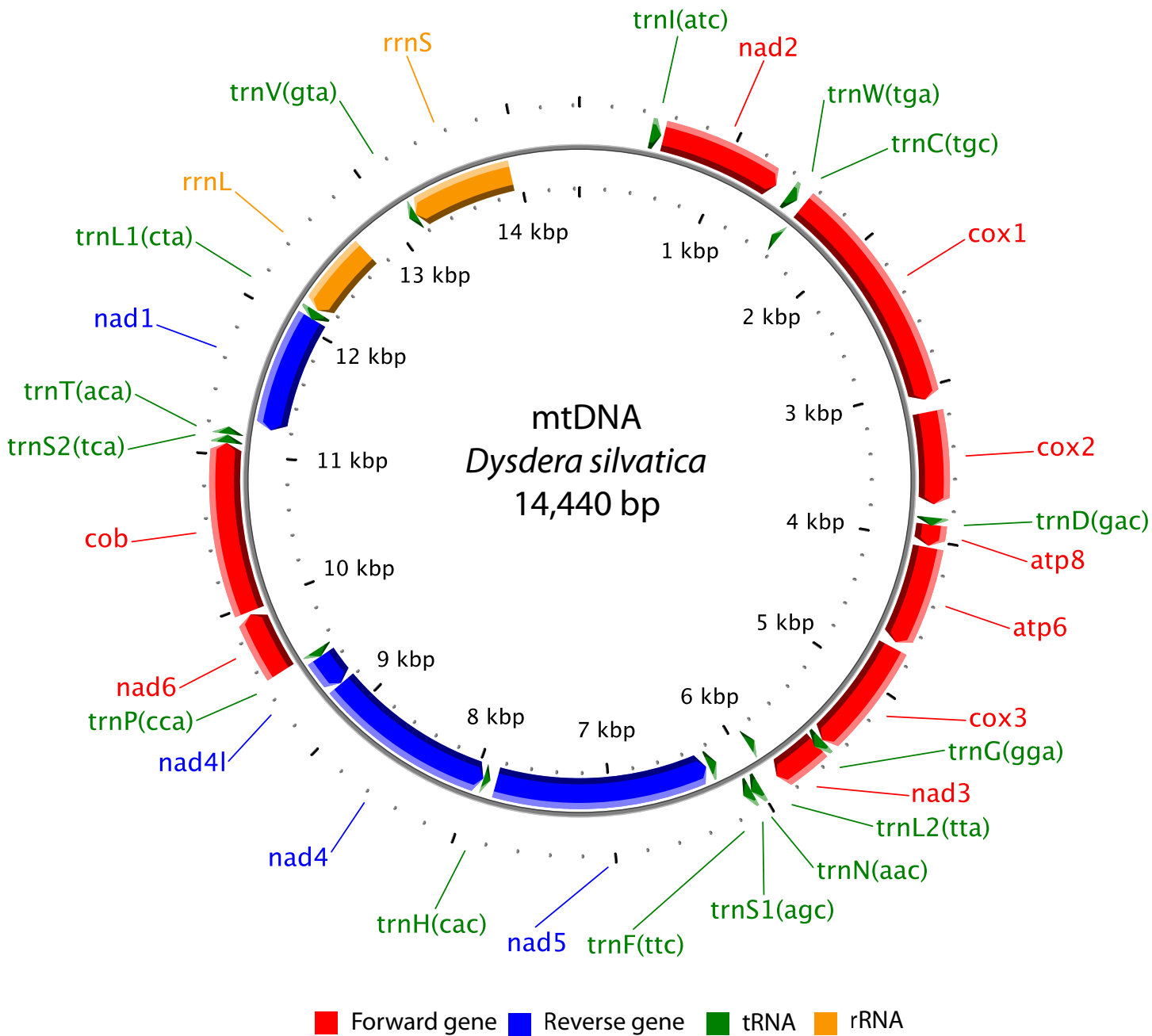

Supplement: giz099_Supplemental_Files [file giz099_supplemental_files.zip › Figure_S10.pdf]

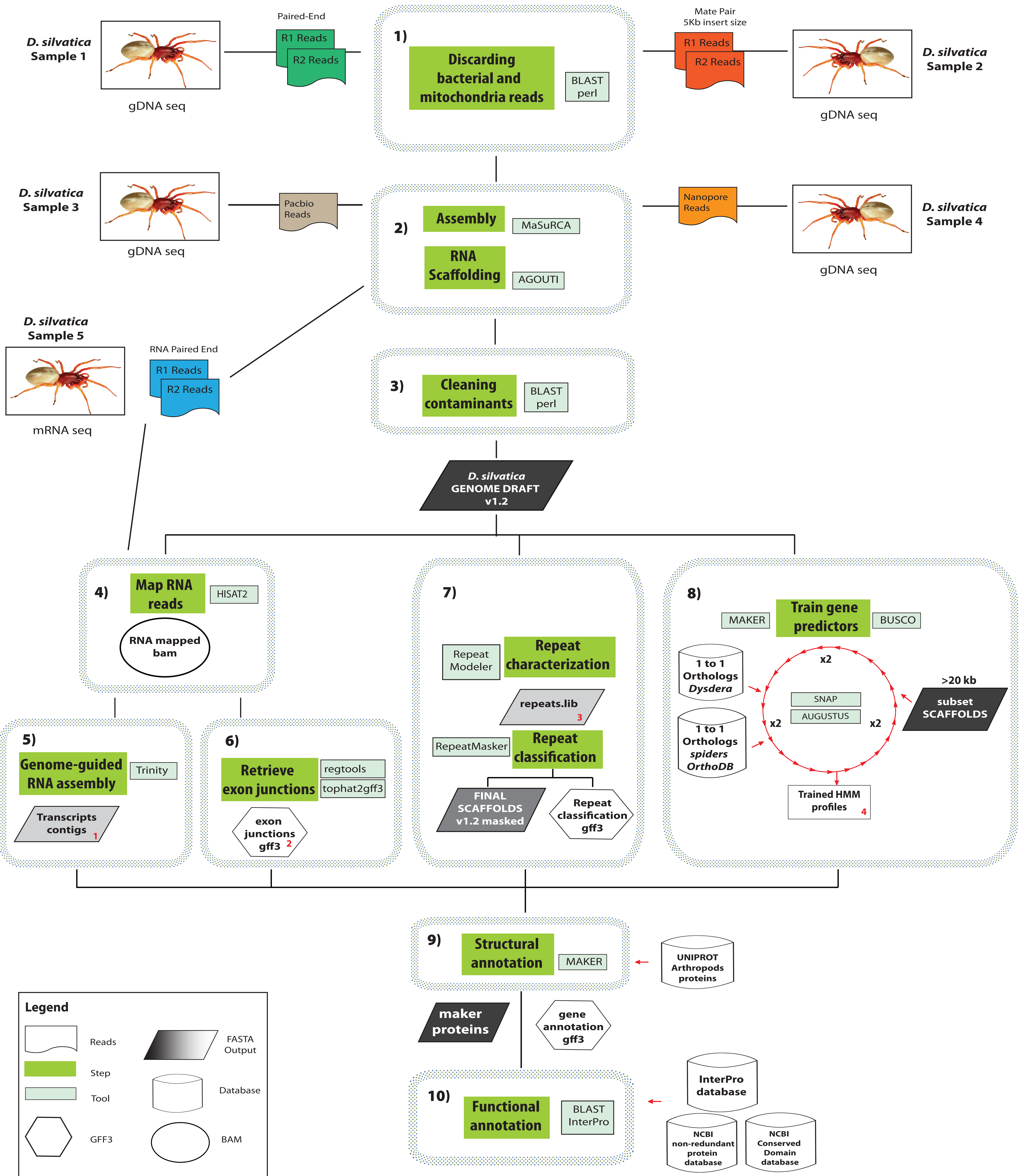

Supplement: giz099_Supplemental_Files [file giz099_supplemental_files.zip › Figure_S2.pdf]

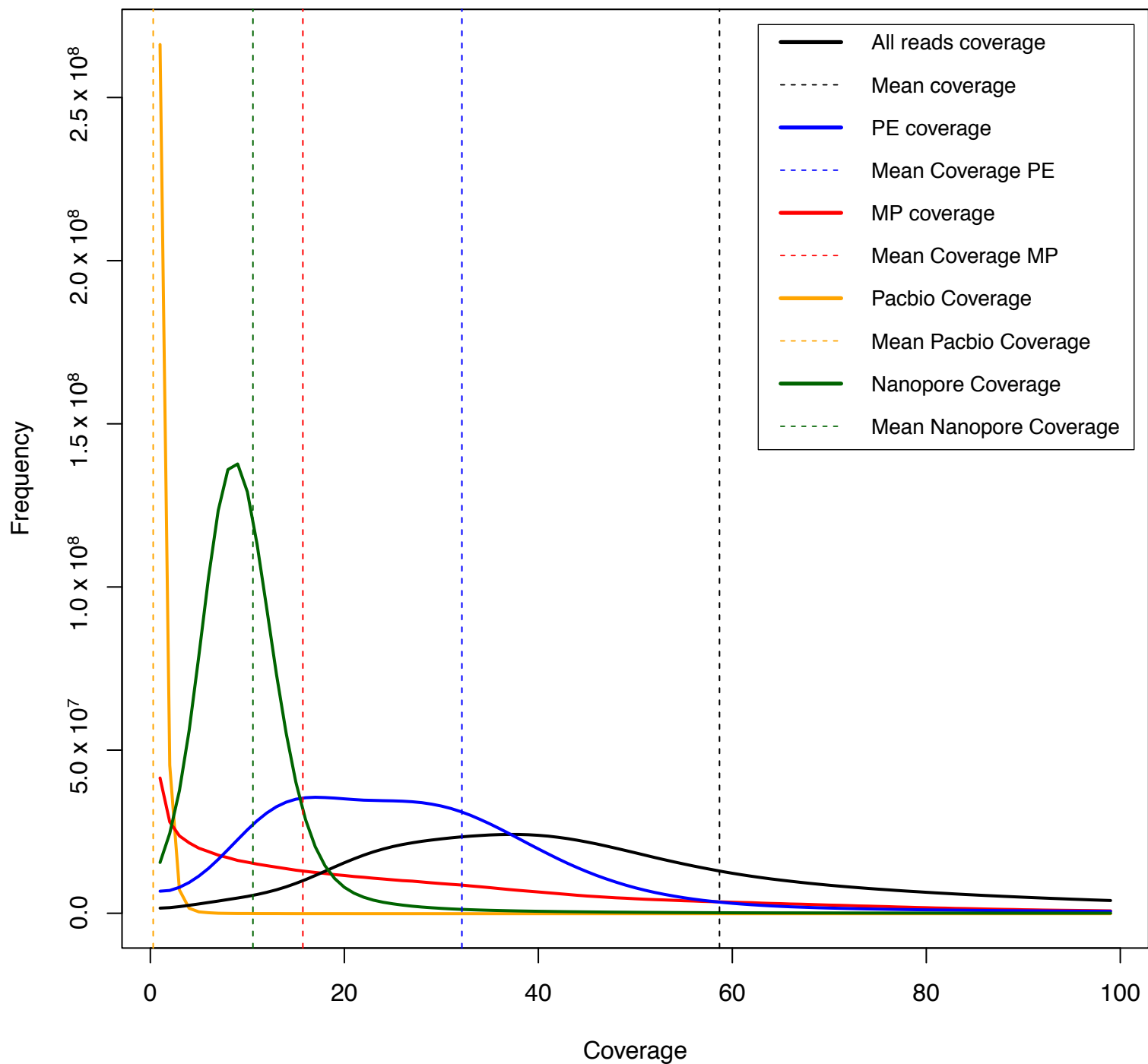

Supplement: giz099_Supplemental_Files [file giz099_supplemental_files.zip › Figure_S3.pdf]

a)

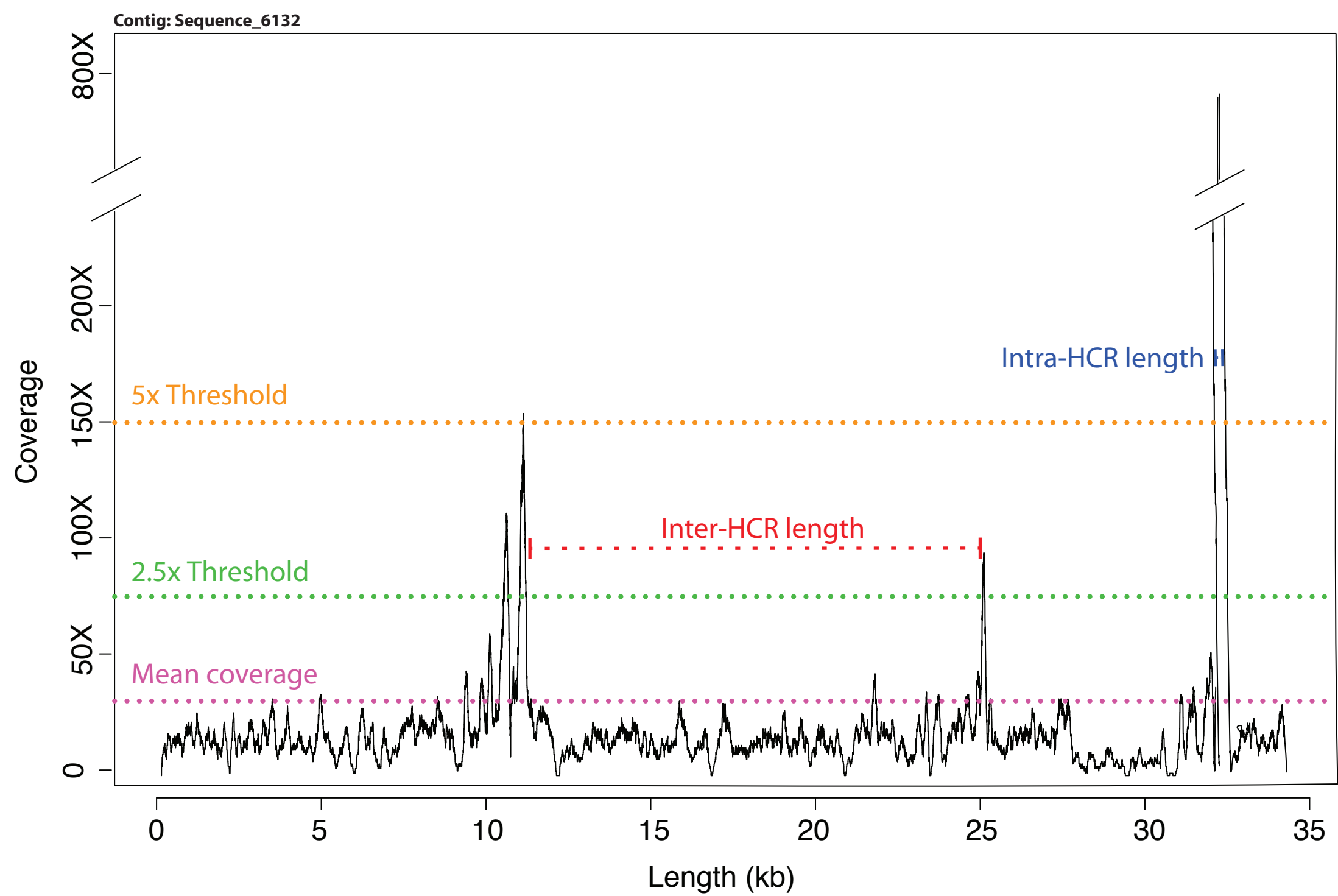

b)

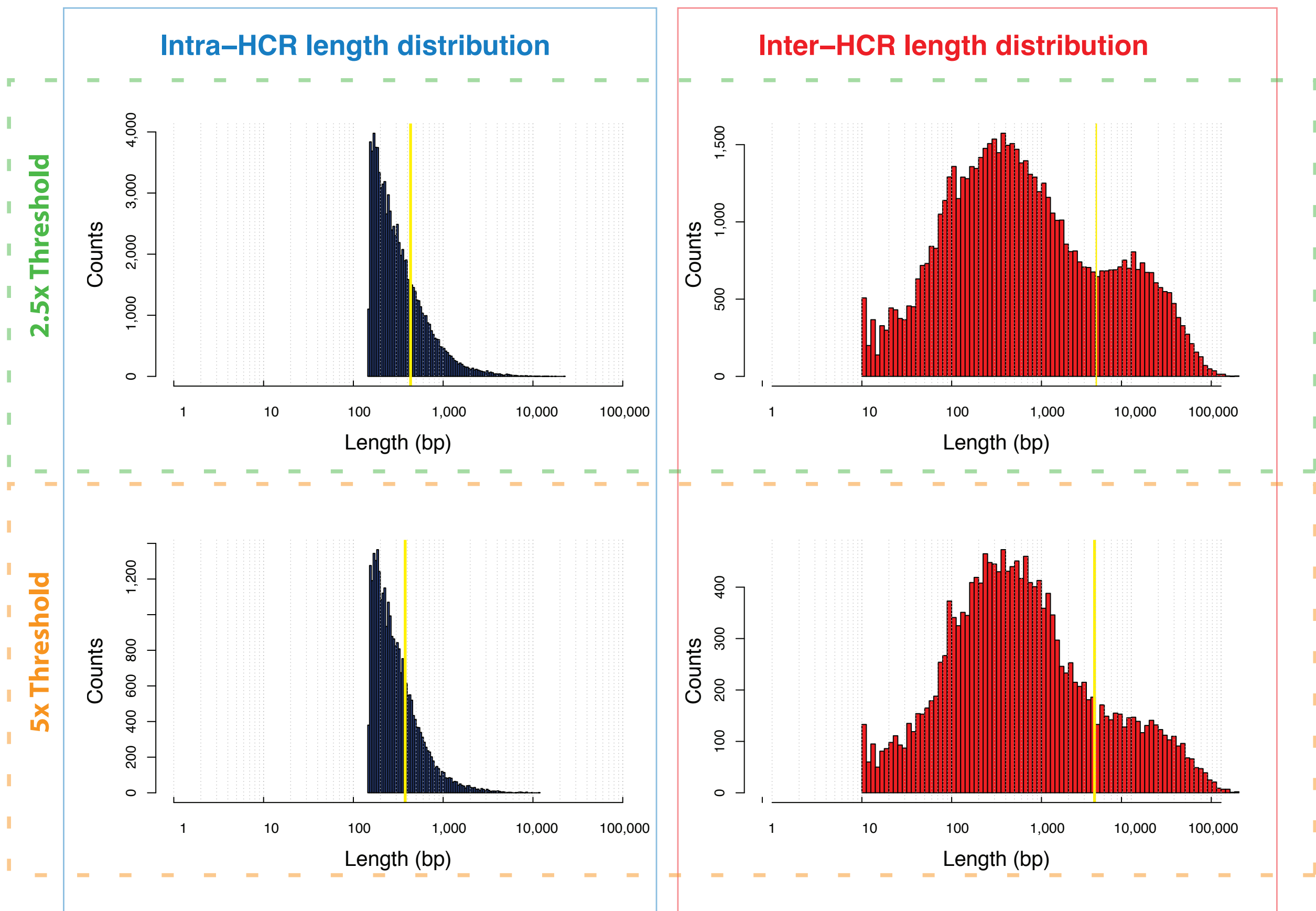

Supplement: giz099_Supplemental_Files [file giz099_supplemental_files.zip › Figure_S4.pdf]

**Intra (blue) and inter (red) HCR distribution for 2.5x Mean coverage**

**> 150 bp**

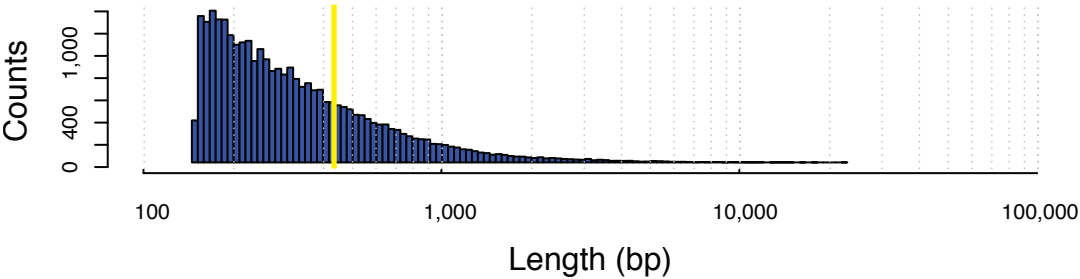

**> 150 bp**

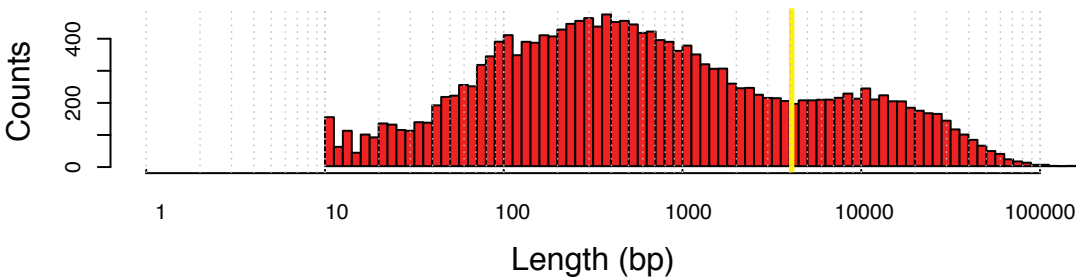

**> 500 bp**

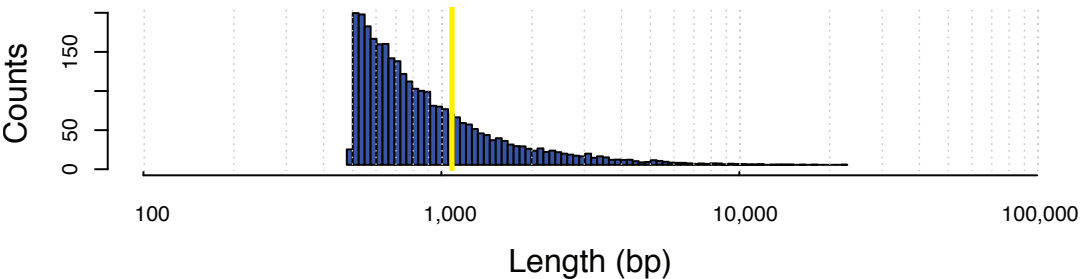

**> 500 bp**

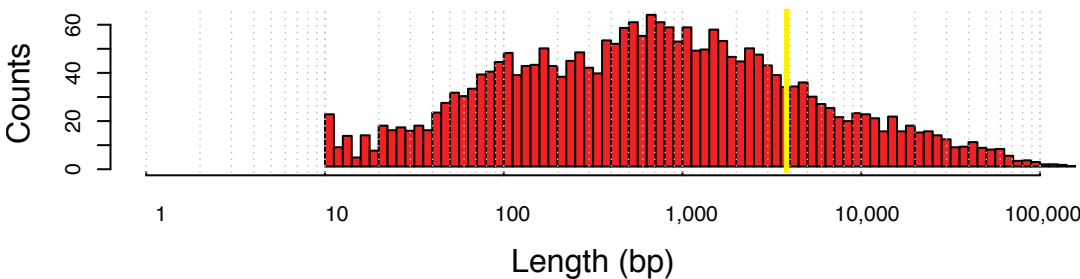

**> 1,000 bp**

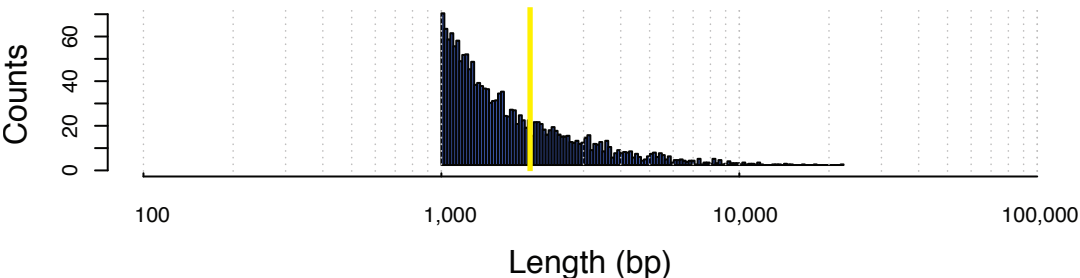

**> 1,000 bp**

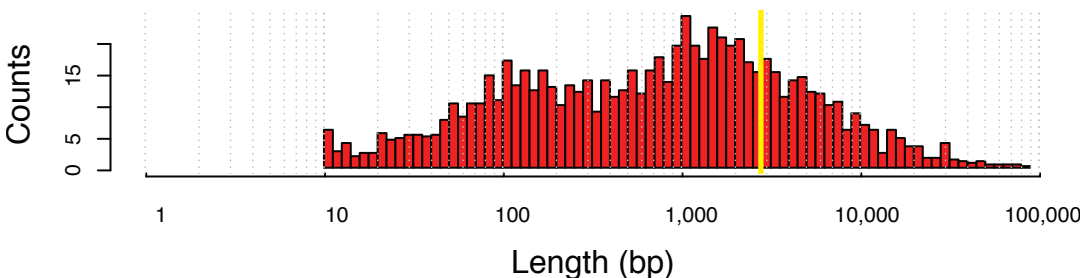

**> 5,000 bp**

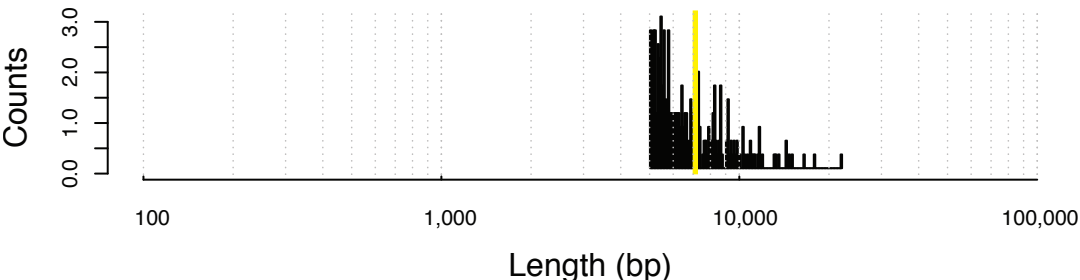

**> 5,000 bp**

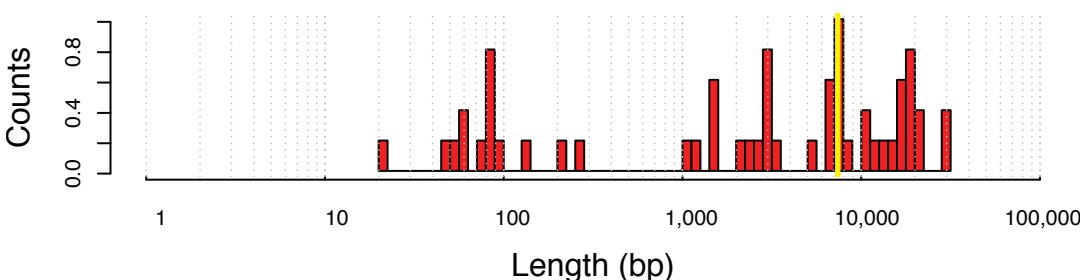

Supplement: giz099_Supplemental_Files [file giz099_supplemental_files.zip › Figure_S5a.pdf]

Intra (blue) and inter (red) HCR distribution for 5x Mean coverage

> 150 bp

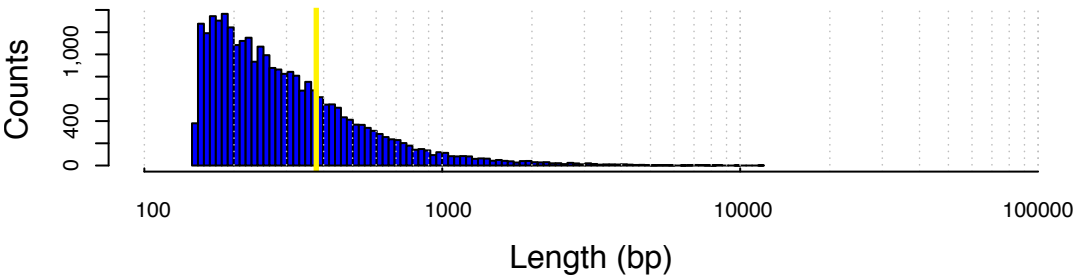

> 150 bp

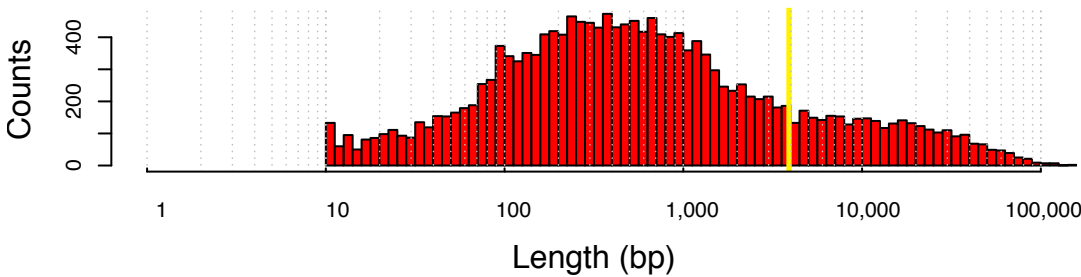

> 500 bp

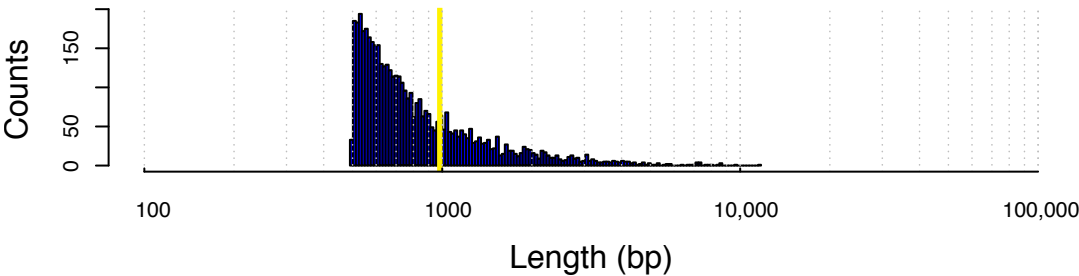

> 500 bp

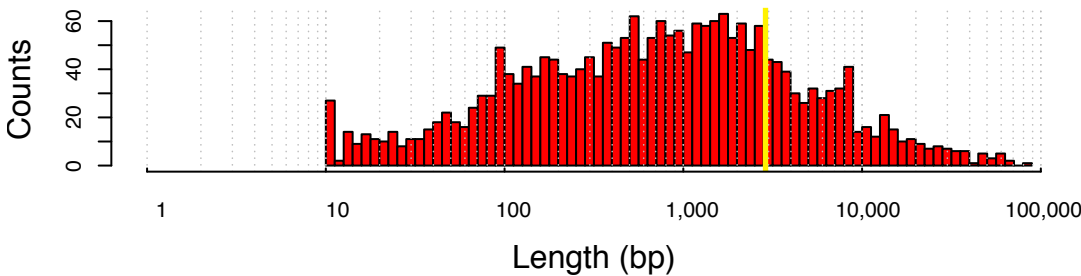

> 1,000 bp

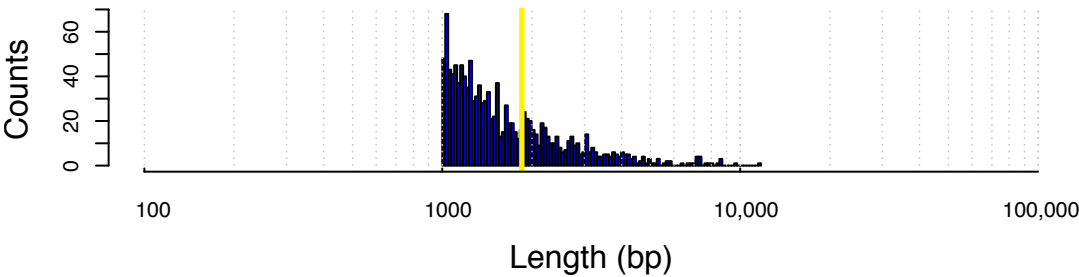

> 1,000 bp

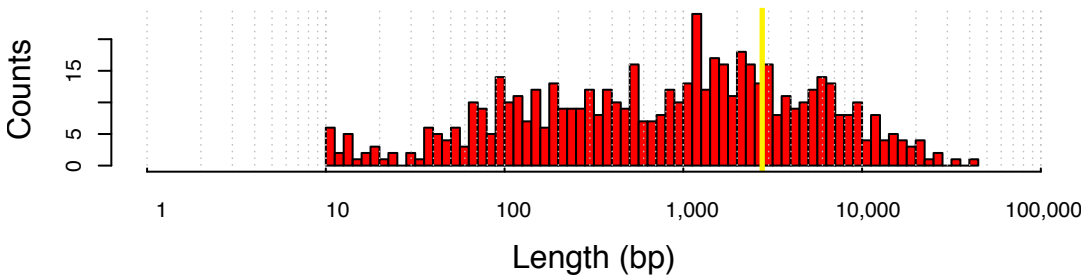

> 5,000 bp

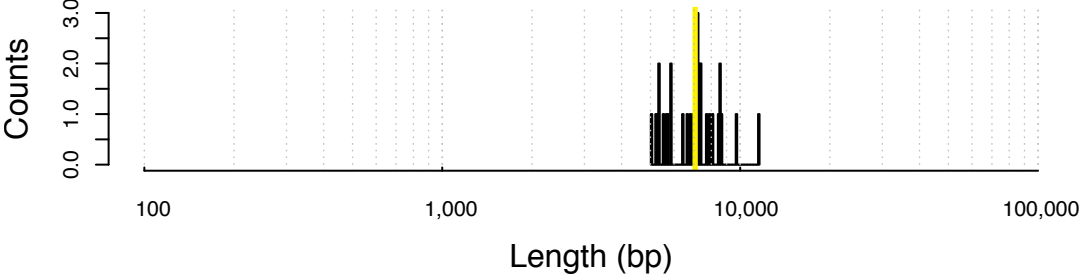

> 5,000 bp

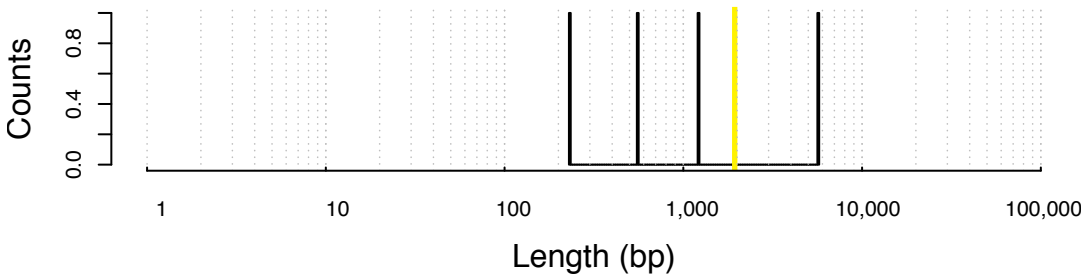

Supplement: giz099_Supplemental_Files [file giz099_supplemental_files.zip › Figure_S5b.pdf]

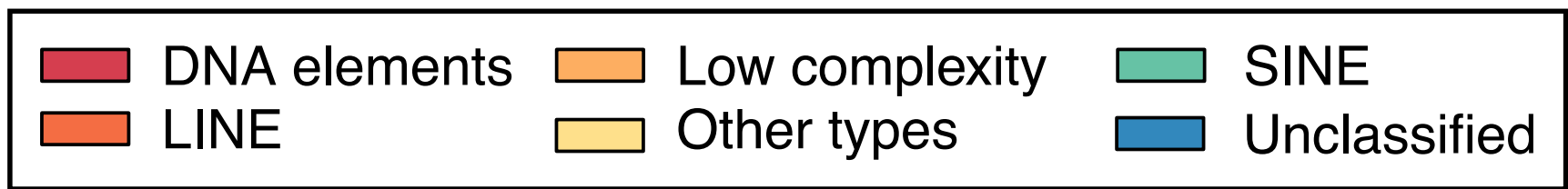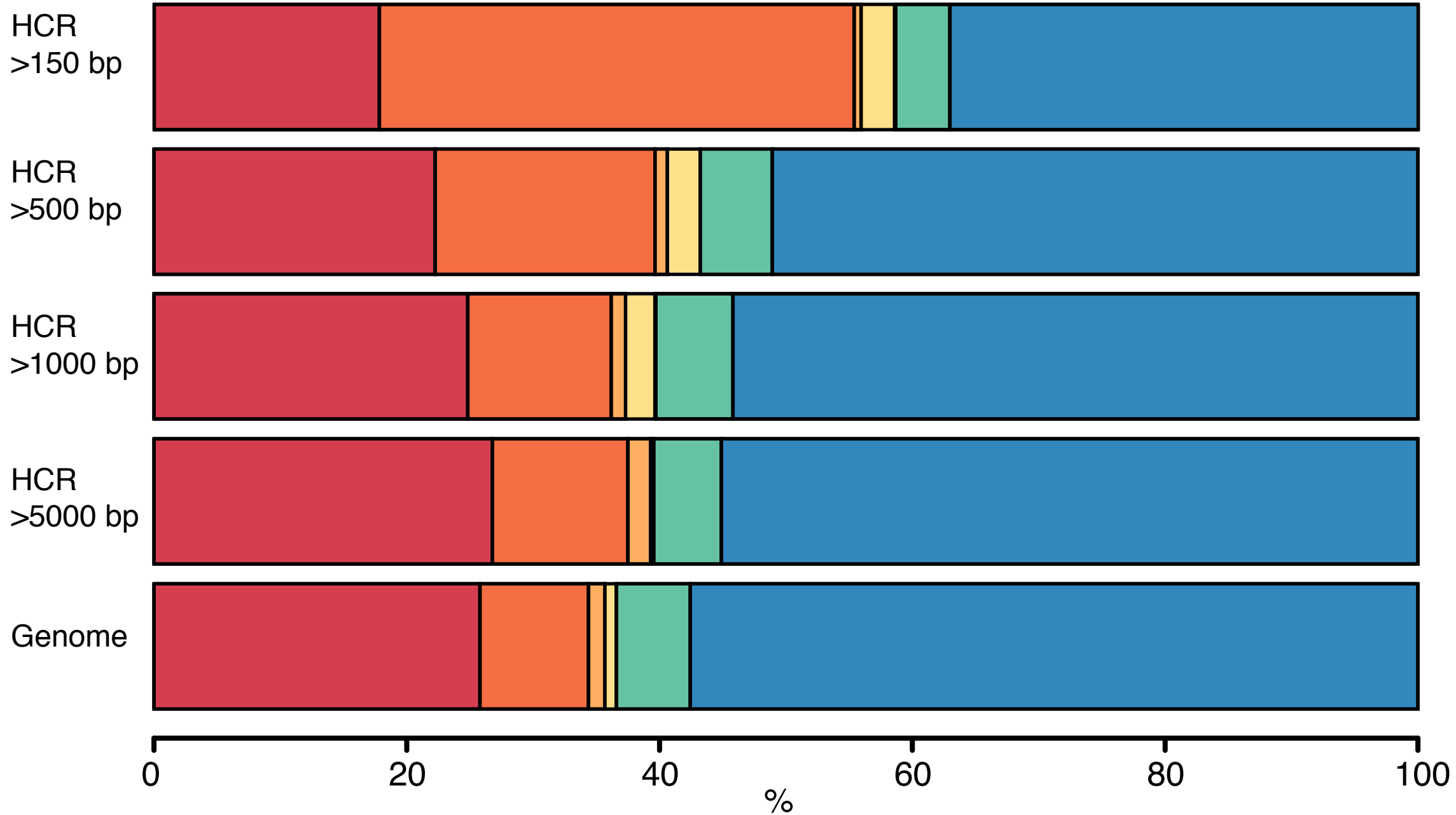

Supplement: giz099_Supplemental_Files [file giz099_supplemental_files.zip › Figure_S6.pdf]

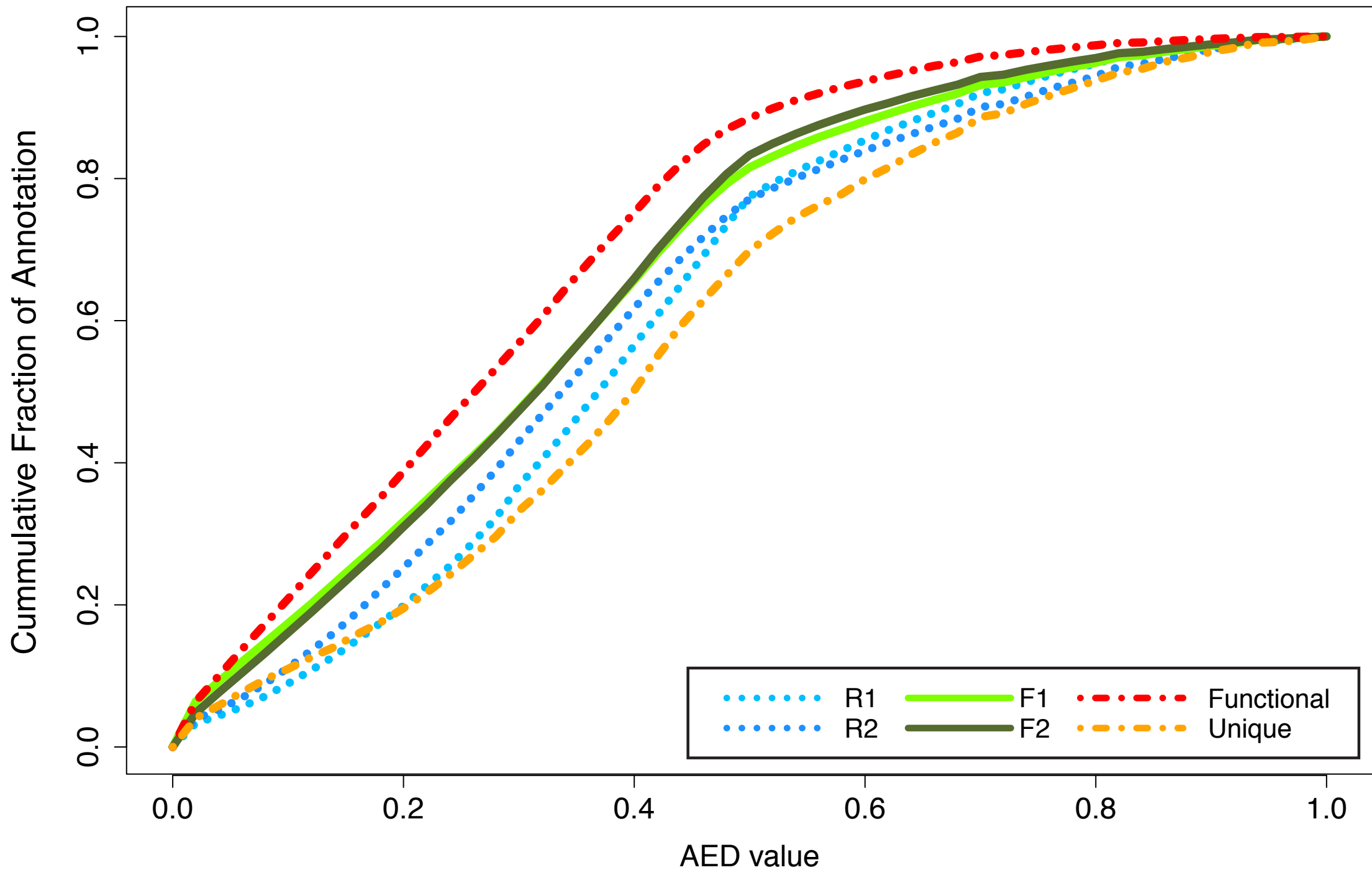

Supplement: giz099_Supplemental_Files [file giz099_supplemental_files.zip › Figure_S7.pdf]

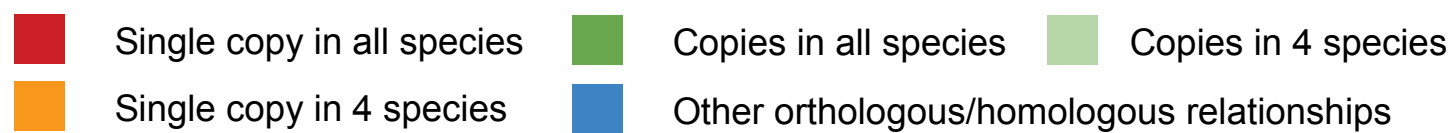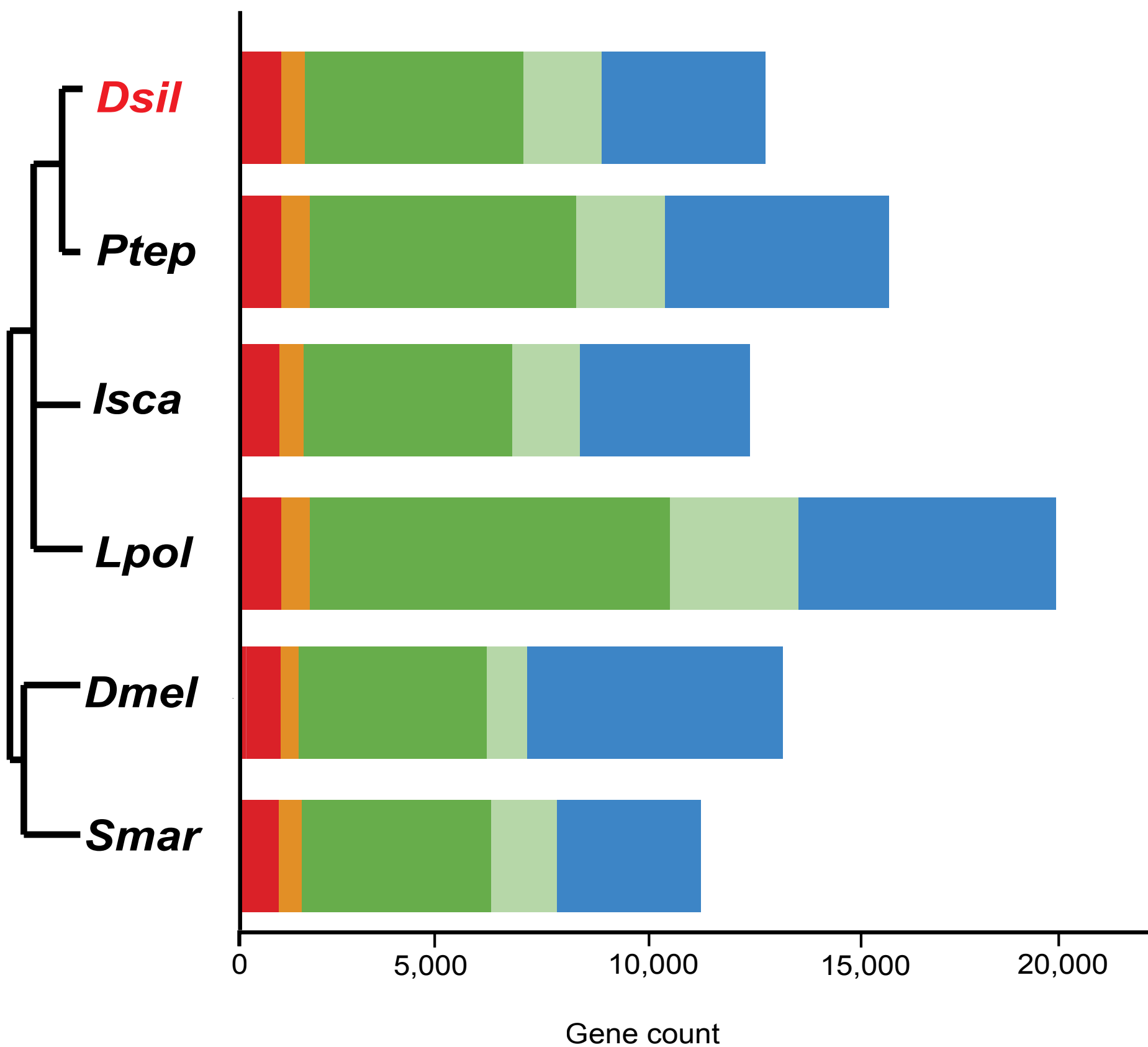

Supplement: giz099_Supplemental_Files [file giz099_supplemental_files.zip › Figure_S8.pdf]

RNA evidence

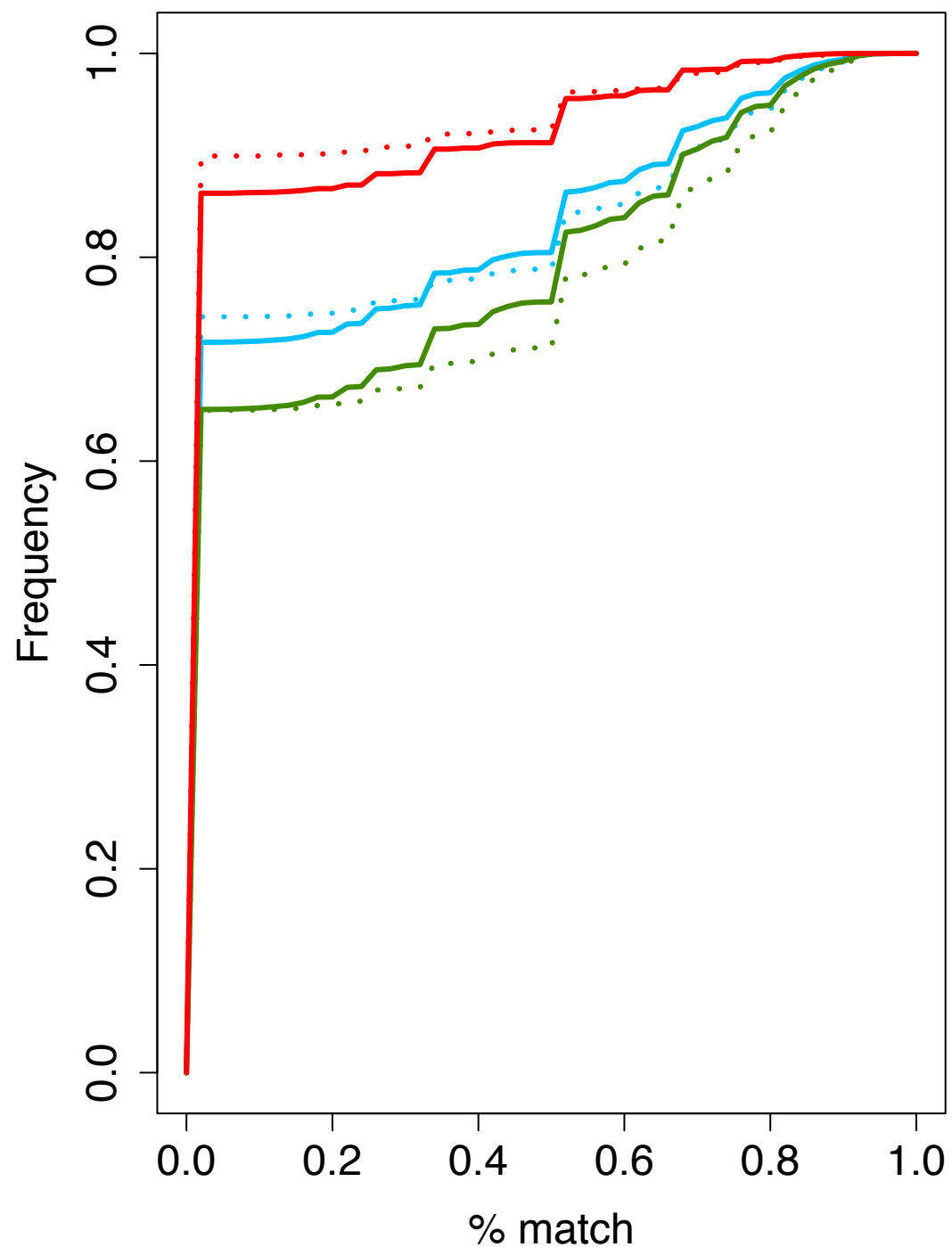*Ab initio* prediction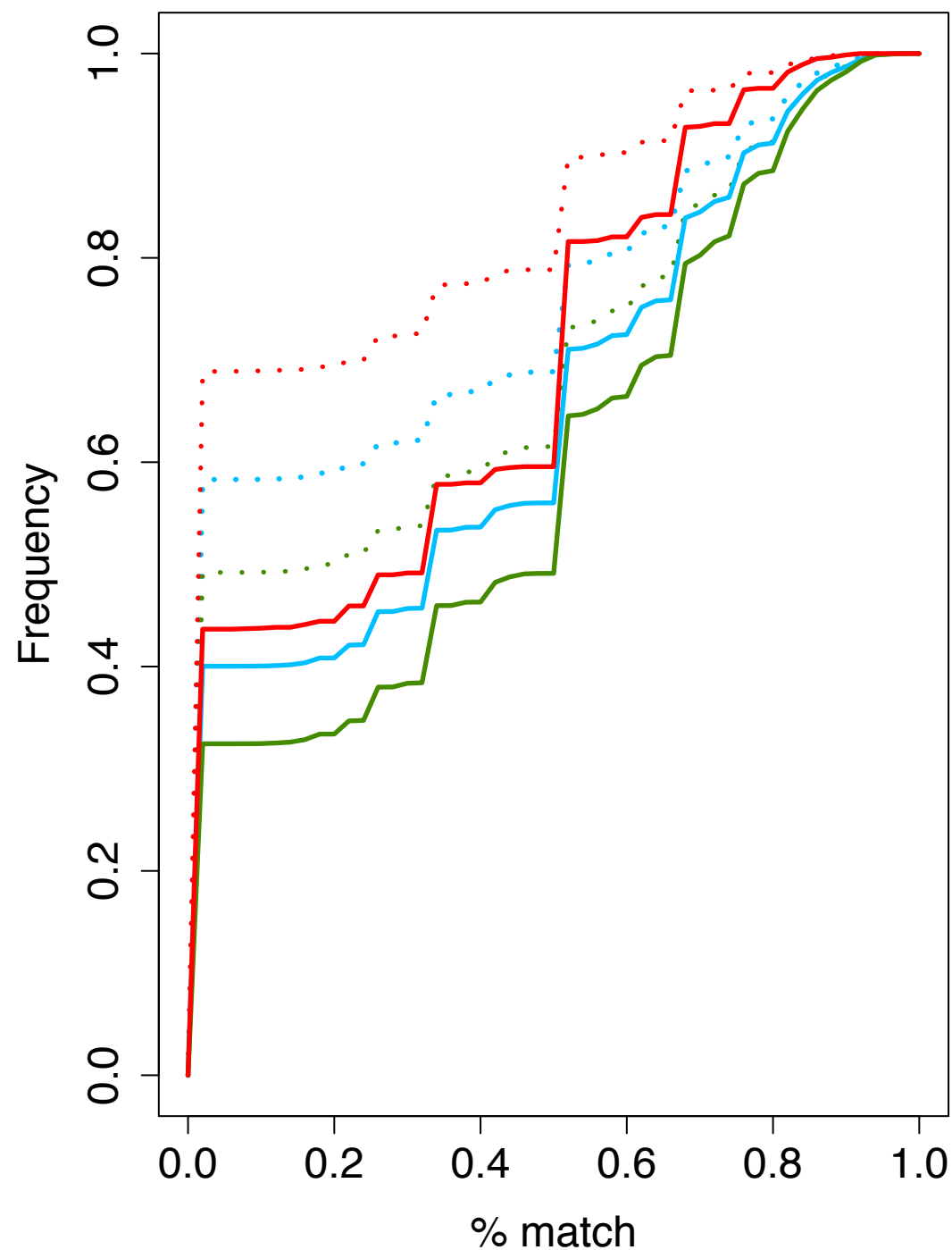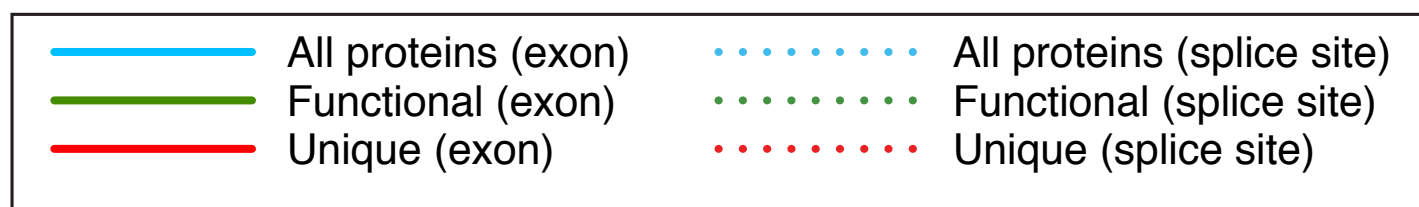

Supplement: giz099_Supplemental_Files [file giz099_supplemental_files.zip › Figure_S9.pdf]
