# Supplementary material for: The draft genome sequence of the spider Dysdera silvatica (Araneae, Dysderidae): A valuable resource for functional and evolutionary genomic studies in chelicerates
Source: Gigascience. 2019 Aug 20;8(8):giz099. doi: 10.1093/gigascience/giz099 (PMC6701490; doi:10.1093/gigascience/giz099)
Supplement: giz099_Response_to_Reviewer_Comments_Original_Submission [file giz099_response_to_reviewer_comments_original_submission.pdf]

Author's Response To Reviewer Comments

Clo<u>s</u>e

Editor Comments to the Author:

Dear Prof. Rozas,

Your manuscript "The draft genome sequence of the spider Dysdera silvatica (Araneae, Dysderidae): A valuable resource for functional and evolutionary genomic studies in chelicerates" (GIGA-D-19-00156) has been assessed by our reviewers. Based on these reports, and my own assessment as Editor, I am pleased to inform you that it is potentially acceptable for publication in GigaScience, once you have carried out some essential revisions suggested by our reviewers.

In addition, please register any new software application in the SciCrunch.org database to receive a RRID (Research Resource Identification Initiative ID) number, and include this in your manuscript. This will facilitate tracking, reproducibility and re-use of your tool.

Since we have no developed any software, we have not used the SciCrunch resource. Nevertheless, we have written a number of scripts to analyze the data. All these scripts are available from the github repository website (the reference is included in the manuscript):
https://github.com/molevol-ub/Dysdera_silvatica_genome

Reviewer Comments to the Author:

Reviewer #1: In the revised manuscript, the authors have addressed the points I previously raised with the original version. I agree the genome assembly has been significantly improved.

There is one concern about Fig. 4b. Approximately, 14,000 protein-coding genes of S. silvatica appear to be analyzed but what are they? This needs to be clarified

The reviewer is right. We included in the GigaScience DB repository (and NCBI repository) the complete information including the structural and functional information of the genes identified in D. silvatica. However, we did not include the information from genes with orthologous-relationships identified by OrthoDB (those in Figures 4b but also in Figure S8). In this updated version, we have included such information in two new supplementary tables, S3-1 and S3-2.

Reviewer #2:
He did not include any comments

Clo<u>s</u>e
