# Supplementary material for: The draft genome sequence of the spider Dysdera silvatica (Araneae, Dysderidae): A valuable resource for functional and evolutionary genomic studies in chelicerates
Source: Gigascience. 2019 Aug 20;8(8):giz099. doi: 10.1093/gigascience/giz099 (PMC6701490; doi:10.1093/gigascience/giz099)
Supplement: giz099_Response_to_Reviewer_Comments_Revision_1 [file giz099_response_to_reviewer_comments_revision_1.pdf]

Author's Response To Reviewer Comments

Close

Dear Sirs,

Please find enclosed the revised version of our manuscript GIGA-D-19-00156R1 entitled “The draft genome sequence of the spider Dysdera silvatica (Araneae, Dysderidae): A valuable resource for functional and evolutionary genomic studies in chelicerates” in which we have fixed some points in your annotated version.

In particular

1. Can you add ORCID identifiers for any authors that have them and RRIDs for software, see:
https://sites.google.com/a/gigasciencejournal.com/gigascience/public-pages/gigascience-rrid-list
Done. Nevertheless, we have be not able to include the ORCID Icon after the researcher author (using your latex template). Now it is a hyperlink
This is the ORCID list, for all authors:

JF Sanchez: https://orcid.org/0000-0001-6771-4807
C. Frias-Lopez: https://orcid.org/0000-0002-8078-9552
P. Escuer: https://orcid.org/0000-0002-5941-0106
S. Hinojosa-Alvarez: https://orcid.org/0000-0001-5802-1697
M. Angel: https://orcid.org/0000-0003-1402-4727
A. Sanchez-Gracia: https://orcid.org/0000-0003-4543-4577
J. Rozas: https://orcid.org/0000-0002-6839-9148

2. In the methods you mention an adapted QIAGEN protocol. As this is probably slightly fiddly do you want to fork and modify the protocols.io protocol and cite the DOI they give you at the appropriate place in the methos? This is probably the protocol to adapt: https://www.protocols.io/view/QIAgen-DNeasy-Blood-Tissue-kit-cultured-cells-dsz6f5
I think there is no need. In fact, we used the original protocol with some very minor adjustments that are also indicated in the paper two lines after (extra wash; one centrifugation step less).

3. Can you cite the GigaDB in the data section at the end of the paper and cite the DOI Mary Ann provided in the references (currently looks like it'd be Ref 66, but if you add the protocol it'll be 67.
Sanchez-Herrero JF; Frías-López C; Escuer P; Hinojosa-Alvarez S; Arnedo MA; Sánchez-Gracia A; Rozas J (2019): Supporting data for "The draft genome sequence of the spider Dysdera silvatica (Araneae, Dysderidae): A valuable resource for functional and evolutionary genomic studies in chelicerates" GigaScience Database. http://dx.doi.org/10.5524/100628
Done

4. For the code you've provided can you also add the following in the availability of supporting materials section:

Availability of supporting source code and requirements
• Project name: Genome assembly of Dysdera silvatica
•
• Project home page: https://github.com/molevol-ub/Dysdera_silvatica_genome
• Operating system(s): Platform independent
• Programming language: Bash, Perl, Python
• Other requirements: e.g. Java 1.3.1 or higher, Tomcat 4.0 or higher

• License: MIT
• RRID: if applicable, e.g. RRID: SCR_014986
Done

In addition, please register any new software application in the SciCrunch.org database to receive a RRID (Research Resource Identification Initiative ID) number, and include this in your manuscript. This will facilitate tracking, reproducibility and re-use of your tool.
Done

With best regards,

Sincerely,

Close
