# Supplementary material for: The draft genome sequence of the spider Dysdera silvatica (Araneae, Dysderidae): A valuable resource for functional and evolutionary genomic studies in chelicerates
Source: Gigascience. 2019 Aug 20;8(8):giz099. doi: 10.1093/gigascience/giz099 (PMC6701490; doi:10.1093/gigascience/giz099)
Supplement: giz099_Response_to_Reviewer_Comments_Revision_2 [file giz099_response_to_reviewer_comments_revision_2.pdf]

Author's Response To Reviewer Comments

Close

Dear Sirs,

Please find enclosed the revised version of our manuscript GIGA-D-19-00156R2 entitled “The draft genome sequence of the spider Dysdera silvatica (Araneae, Dysderidae): A valuable resource for functional and evolutionary genomic studies in chelicerates” in which we have fixed some points in your annotated version.

In particular

For the numbers in the text and tables, you wrote like this "39 609 522 995", please replace the space with comma in all numbers.
Done

And you can also revise the following things this time:
1) RRIDs for software: eg. you wrote "with Jellyish v.2.2.3 (RRID:SCR_005491) [28].", it should be "with Jellyish v.2.2.3 (Jellyish, RRID:SCR_005491) [28]." Please revise all these writings.
Done

2) For the QIAGEN protocol issue Scott mentioned in last email, if you think it's a better way to present the protocol, you can add it this time.
I think there is no need. We have used the original protocol of QIAGEN, with some very minor adjustments that are also indicated in the paper (extra wash; one centrifugation step less). Moreover, there is no any entry in protocols.io for the original QUIAGEN.

With best regards,

Julio Rozas

Close
