# Supplementary material for: The draft genome sequence of the spider Dysdera silvatica (Araneae, Dysderidae): A valuable resource for functional and evolutionary genomic studies in chelicerates
Source: Gigascience. 2019 Aug 20;8(8):giz099. doi: 10.1093/gigascience/giz099 (PMC6701490; doi:10.1093/gigascience/giz099)
Supplement: giz099_Response_to_Reviewer_Comments_Revision_3 [file giz099_response_to_reviewer_comments_revision_3.pdf]

Author's Response To Reviewer Comments

Close

I am sending the revised PDF, including your suggested changes.

Close
